# HLA‐DR3 mediated CD4 T cell response against GAD65 in type 1 diabetes patients

**DOI:** 10.1111/1753-0407.13406

**Published:** 2023-06-13

**Authors:** Neihenuo Chuzho, Neetu Mishra, Nikhil Tandon, Uma Kanga, Gunja Mishra, Akanksha Sharma, Narinder K. Mehra, Neeraj Kumar

**Affiliations:** ^1^ Indian Council of Medical Research (ICMR)‐National Institute of Pathology Safdarjung Hospital Campus New Delhi India; ^2^ Symbiosis School of Biological Sciences Symbiosis International (Deemed University) Pune India; ^3^ Department of Endocrinology and Metabolism All India Institute of Medical Sciences New Delhi India; ^4^ Department of Transplant Immunology and Immunogenetics All India Institute of Medical Sciences New Delhi India; ^5^ Emeritus Scientist (ICMR), and Former Dean (Research) All India Institute of Medical Sciences New Delhi India

**Keywords:** CD4 T cells, cytokines, GAD65, HLA, type 1 diabetes, CD4 T细胞, 细胞因子, GAD65, HLA, 1型糖尿病

## Abstract

**Aim:**

We planned this study to identify diabetogenic glutamic acid decarboxylase (GAD65) peptides possibly responsible for human leucocyte antigen (HLA)‐DR3/DQ2‐mediated activation of GAD65‐specific CD4 T cells in type 1 diabetes (T1D).

**Methods:**

Top 30 GAD65 peptides, found to strongly bind in silico with HLA‐DR3/DQ2 molecules, were selected and grouped into four pools. The peptides were used to stimulate CD4 T cells of study subjects in 16‐h peripheral blood mononuclear cell culture. CD4 T cells' stimulation in terms of interferon‐gamma (IFN‐γ), interleukin (IL)‐17, tumor necrosis factor‐alpha (TNF‐α), and IL‐10 expression was analyzed using flow cytometry.

**Results:**

Although all four GAD65 peptide pools (PP1‐4) resulted in significantly higher expression of IFN‐γ by CD4 T cells (*p* = .003, *p* < .0001, *p* = .026, and *p* = .002, respectively), only pool 2 showed significant increase in IL‐17 expression (*p* < .0001) in T1D patients vs healthy controls. Interpeptide group comparison for immunogenicity revealed significantly higher IFN‐γ and IL‐17 expressions and significantly lower IL‐10 expression for PP2 compared to other groups (*p* < .0001, *p* = .02, and *p* = .04, respectively) in patients but not in controls. Further, group 2 peptides resulted in significant increase in CD4 T cells' expression of IFN‐γ and IL‐17 (*p* = .002 for both) and significant decrease in IL‐10 (*p* = .04) in HLA‐DRB1*03‐DQA1*05‐DQB1*02+ patients vs HLA‐DRB1*03‐DQA1*05‐DQB1*02+ controls. The CD4 T cells' expression of IL‐17 was significantly higher (*p* = .03) in recently diagnosed vs long‐standing HLA‐DRB1*03‐DQA1*05‐DQB1*02+ T1D patients.

**Conclusion:**

GAD65 peptides, particularly those belonging to PP2, induced CD4 T cells to express IFN‐γ and IL‐17 cytokines in T1D patients, suggesting that group 2 peptides possibly presented by HLA‐DR3 molecule to CD4 T cells shift immune balance toward inflammatory phenotype in patients.

## Graphical abstract

**Highlights**

- The CD4 T cells of type 1 diabetes (T1D) patients particularly having HLA‐DRB1*03‐DQA1*05‐DQB1*02 haplotype are reactive against a particular region of the glutamic acid decarboxylase (GAD65) protein.
- GAD65 peptides encompassing 288–308 amino acid positions (peptide pool 2) may be favored by the HLA‐DR3 molecule for presentation to CD4 T cells in T1D.
- Interleukin (IL)‐17 producing CD4 T cells (Th17) possibly have an important role in driving inflammatory CD4 T cell response in T1D.
- Two cytokines, IFN‐γ and IL‐17, might have a key role in HLA‐DR3 mediated CD4 T cell immune response to GAD65 protein in T1D.

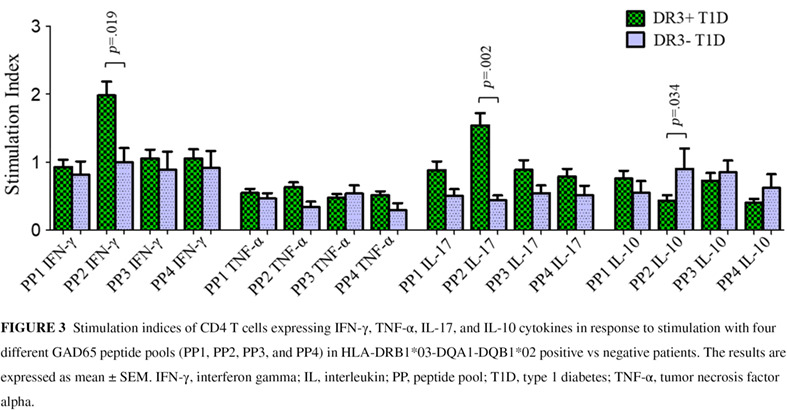

## 摘要

目的:我们进行这项研究以确定与1型糖尿病(T1D)中人类白细胞抗原(HLA)‐DR3/DQ2介导的谷氨酸脱羧酶(GAD65)特异性CD4 T细胞激活可能相关的致糖尿病性GAD65肽。

方法:选取与HLA‐DR3/DQ2分子在体外结合强烈的前30个GAD65肽, 分为4个池组。使用这些肽来刺激研究对象的CD4 T细胞, 在16小时PBMC培养中分析CD4 T细胞对干扰素(IFN‐γ), 白介素(IL)‐17, 肿瘤坏死因子‐α(TNF‐α)和IL‐10的激活表达, 使用流式细胞术进行分析。

结果:所有四个GAD65肽池(PP1‐4)都导致CD4 T细胞的IFN‐γ表达明显增加(p=0.003, p<0.0001, p=0.026 和 p=0.002, 分别), 只有池2在T1D患者与健康对照组之间显示出显著的IL‐17表达增加(p<0.0001)。肽间组间比较显示, 在患者中, PP2组与其他组相比, IFN‐γ和IL‐17的表达显著增加, IL‐10的表达显著降低(p<0.0001, p=0.02 和 p=0.04), 但在对照组中没有显示出明显差异。此外, 2号组肽对于HLA‐DRB1 * 03‐DQA1 * 05‐DQB1 * 02 + 患者而言, 导致CD4 T细胞的IFN‐γ和IL‐17表达显著增加(p=0.002), 而IL‐10表达显著降低(p=0.04)。在HLA‐DRB1 * 03‐DQA1 * 05‐DQB1 * 02 +的最近诊断的T1D患者中, CD4 T细胞的IL‐17表达显著增加(p=0.03)。

结论:GAD65肽, 尤其是属于PP2的肽段, 在T1D患者中能够诱导CD4 T细胞表达IFN‐γ和IL‐17细胞因子, 这表明在患者中2号组肽可能通过HLA‐DR3分子递呈给CD4 T细胞, 使免疫平衡朝向炎症表型转变。

## 1 INTRODUCTION

Type 1 diabetes (T1D) is a complex autoimmune disease characterized by the presence of autoantibodies against β‐cell antigens and T cell mediated destruction of the islet β‐cells.^1^, ^2^ Susceptibility to T1D is strongly linked to genetic factors, particularly, the human leucocyte antigen (HLA) region on chromosome 6p21.31.^3^ During normal immune response, fragments of antigenic peptides are carried by the HLA class II molecules to the surface of antigen presenting cells (APCs) and presented to the CD4 T cells. A key element of T1D pathogenesis is the presentation of islet β‐cell antigens by HLA‐DR molecule, HLA‐DQ molecule, or both to CD4 T cells resulting in autoimmune response against islet β‐cells, leading to their loss and lifelong dependence on exogenous insulin for T1D patients.^4^, ^5^ An array of β‐cells‐specific proteins have been identified as autoimmune targets by screening for the presence of autoantibodies against diabetes‐associated antigens in sera of prediabetic and diabetic patients. Some common T1D‐associated autoantigens are insulin, glutamic acid decarboxylase‐65 (GAD65), islet antigen‐2 (IA‐2), and zinc transporter‐8 (ZnT8).^1^, ^6^ Among these, autoantibody against GAD65 is one of the most important serological biomarkers as it is reported to be present in 70%–80% of prediabetic and early‐onset recent T1D patients.^7^, ^8^ Studies have also reported CD4 T cell responses to synthetic peptides of GAD65 in T1D patients and its animal models.^4^, ^9^, ^10^, ^11^ Splenic T lymphocytes from nonobese diabetic (NOD) mice, a convenient animal model for human T1D, spontaneously proliferated when incubated with GAD65 from humans, showing GAD65 to be a T cell antigen in NOD mice.^12^ Adoptive transfer of T cells specific for single diabetogenic peptide triggered the onset of T1D in NOD mice.^9^ Further, the immunization with GAD65 peptides have been reported to generate Th2 and Tr1 cells and prevent T1D in NOD mice.^13^ Based on the animal studies investigations have been carried out to understand the immune response of CD4 T cell to diabetogenic peptides in humans. This yielded varying outcomes as the development of T1D is a dynamic process and is influenced by diverse environmental and genetic factors. For instance, vaccination with GAD65‐alum in humans was reported to preserve the residual insulin secretion in recent‐onset T1D patients, although it did not change the insulin requirement.^14^

Immunogenetic studies have reported a strong association of HLA‐DRB1*03‐DQA1*05‐DQB1*02 (DR3‐DQ2) haplotype with North Indian T1D patients.^15^, ^16^, ^17^, ^18^ Further, studies revealed that glutamic acid decarboxylase 65 autoantibody (GADA) is also associated with HLA‐DRB1*03 and DQB1*02 alleles in T1D patients, suggesting an important role of these HLA class II alleles in the generation of GADA.^19^, ^20^, ^21^ However, the exact mechanism behind these genetic associations remains elusive and, therefore, it is of great interest to identify diabetogenic GAD65 peptides and their role in HLA‐DR3‐DQ2 mediated autoimmune response in T1D. Further, it is not known whether there is a difference in the T cell response against the diabetogenic peptides, particularly that of CD4 T cells, between T1D patients and healthy controls (HCs) carrying HLA‐DRB1*03‐DQA1*05‐DQB1*02 haplotype. Therefore, we aimed to study the HLA‐DR3/DQ2 mediated CD4 T cell response against GAD65 in study subjects using overlapping GAD65 peptides that were predicted in silico to bind strongly to the high risk‐DR3/DQ2 molecules. We evaluated the GAD65 peptides possibly presented to CD4 T cells by the HLA‐DR3 molecule, HLA‐DQ2 molecule, or both in T1D patients and how do these differ from that in healthy persons?

## 2 MATERIALS AND METHODS

### 2.1 Study subjects and approvals

One hundred and ten (110) T1D patients, clinically diagnosed as per the criteria described under the Registry of Youth Onset Diabetes in India,^19^, ^22^ were recruited from Out Patients Department/Diabetes of the Young (DOY) Clinic, Department of Endocrinology and Metabolism, All India Institute of Medical Sciences, New Delhi, India. This study did not include prediabetic individuals and all patients who were part of this study were already on exogenous insulin therapy for diabetes management. Persons with other forms of diabetes such as latent autoimmune diabetes in adults, gestational diabetes mellitus, etc., were also excluded from this study. Further, 127 sex‐ and age‐matched healthy controls (HC) were enrolled in the study as controls. All the study participants belonged to North India region, that is, Haryana, Himachal Pradesh, Punjab, Uttarakhand, Uttar Pradesh and the union territories of Jammu and Kashmir, Chandigarh, and Delhi. The ethical approval for this study was granted by the Institutional Ethics Committee. An informed consent was obtained from each study subject (or legal guardian if the patient was a minor) before participation in the study.

The demographic analysis of the study population is given in Table 1. Out of 110 type 1 diabetes patients, 57 (51.82%) were male and 53 (48.18%) were female. Further, 67 (52.76%) out of 127 healthy controls were male and 60 (47.24%) were female. The median age of patients at T1D onset and disease duration in our study was 11 years and 6 years, respectively. The median age of patients at sampling was 20 years and that of HC was 23 years (Table 1). We further categorized the T1D patients on the basis of age at T1D onset and disease duration and HC group based on their age, as shown in Figure S1 in Data S1.

**TABLE 1:** Demographic particulars of 110 T1D patients and 127 healthy controls (HC) from North India.

Phenotype		Study subjects	
		T1D patients	HC
Total number of subjects, *N* (%)		110	127
Median age‐at‐disease onset in years (range)		11 (6 m‐30)	NA
Median age‐at‐sampling in years (range)		20 (2–50)	23 (7–56)
Median disease duration in years (range)		6 (3 m‐31)	NA
Median HbA1c level (range)		7.65 (5.8–11.7)	NA
Sex	Male (%)	57 (51.82)	67 (52.76)
	Female (%)	53 (48.18)	60 (47.24)

Abbreviations: HbA1c, glycated hemoglobin; HC, healthy controls; m, month(s); NA, data not available or applicable; T1D, type 1 diabetes.

At a single time point, 10 mL of peripheral blood was collected from each study subject using a single‐use hypodermic syringe with a 21G needle. The blood was immediately divided into three parts: (a) 3 mL of blood was transferred to EDTA‐coated vials and used for DNA extraction for HLA typing, (b) 2 mL of blood was transferred to serum separation tube to obtain serum for GAD65 autoantibody ELISA assay, and (c) 5 mL of blood was transferred to heparin‐coated vials, which was used to isolate peripheral blood mononuclear cells (PBMC) for T cell stimulation assay and flow cytometry.

### 2.2 Extraction of DNA and HLA‐DRB1/DQA1/DQB1 typing

The DNA of study samples was extracted using the manual salting‐out method, as described previously.^23^ The extracted DNA was washed in 70% ethanol, dried in air and dissolved in Tris‐EDTA buffer (pH = 8.0) and quantified using spectrophotometry. A low‐resolution HLA class II genotyping (HLA‐DRB1, DQA1, and DQB1) was done using a commercially available HLA typing kit, the Hysto Type SSP DR/DQ Combi Pack genotyping kit (BAG Healthcare, Germany). Sequence‐specific primers‐polymerase chain reaction (SSP‐PCR) was carried out using the prealiquoted primers in a 32‐welled plate. The PCR was performed as per the instructions of the kit manufacturer. The PCR‐amplified product was subjected to electrophoresis on a 2% agarose gel and the HLA class II alleles were assigned by using the table for allele interpretation that was provided along with the kit.

### 2.3 GADA ELISA

For GADA screening, we used commercially available ELISA assay kit, ElisaRSR™GADAb kit (RSR Ltd., United Kingdom). The GADA was estimated by following the protocol as described previously.^19^

### 2.4 PBMC isolation

The widely used density gradient centrifugation method was used to obtain PBMC. We used Lymphoprep™ (Stemcell Technologies, Canada) as the density gradient medium to separate out the PBMC layer from the other components of the blood. The PBMC layer was pipetted out into a fresh tube and washed thrice with 1xPBS (Vivantis, Malaysia), resuspended in 1 mL of RPMI 1640 medium supplemented with 10% FBS (Gibco, USA), and then counted using hemocytometer.

### 2.5 Prediction and selection of GAD65 peptides

An online epitope prediction and analysis tool (http://tools.immuneepitope.org) was used to predict GAD65 peptides that were found to bind in silico to HLA‐DR3 (DRB1*03:01) and HLA‐DQ2 (DQA1*05:01‐DQB1*02:01) molecules. From the predicted list, the top 30 GAD65 overlapping peptides were selected based on the percentile rank (the lower the percentile rank, higher the probability of binding) (Table 2). Each peptide was dissolved in suitable solvent (double distilled water or DMSO, dimethyl sulfoxide) at a peptide concentration of 10 mg/mL. Four peptide pools (PP), consisting of 7–9 peptides based on their amino acid positions, were prepared by mixing equal amount of each peptide at a final concentration of 10 μg/mL/peptide (Table 2).

**TABLE 2:** List of top 30 peptides that were predicted to bind in silico to HLA‐DR3 (DRB1*03:01) and HLA‐DQ2 (DQA1*05:01‐DQB1*02:01) using an online epitope prediction tool. The peptides were grouped into four peptide pools (PP1–4) according to their amino acid positions.

Sl. No.	HLA Allele	GAD65 peptide	Start	End	Percentile rank	Peptide pool
1	DRB1*03:01	TDSVILIKCDERGKM	296	310	0.09	PP1
2	DRB1*03:01	DSVILIKCDERGKMI	297	311	0.09	
3	DRB1*03:01	SVILIKCDERGKMIP	298	312	0.1	
4	DRB1*03:01	VILIKCDERGKMIPS	299	313	0.1	
5	DRB1*03:01	ILIKCDERGKMIPSD	300	314	0.1	
6	DRB1*03:01	LIKCDERGKMIPSDL	301	315	0.68	
7	DRB1*03:01	IKCDERGKMIPSDLE	302	316	0.73	
8	DRB1*03:01	GAAALGIGTDSVILI	288	302	0.22	PP2
9	DRB1*03:01	AAALGIGTDSVILIK	289	303	0.22	
10	DRB1*03:01	AALGIGTDSVILIKC	290	304	0.22	
11	DRB1*03:01	ALGIGTDSVILIKCD	291	305	0.22	
12	DRB1*03:01	LGIGTDSVILIKCDE	292	306	0.22	
13	DRB1*03:01	GIGTDSVILIKCDER	293	307	0.26	
14	DRB1*03:01	IGTDSVILIKCDERG	294	308	0.26	
15	DRB1*03:01	NREGYEMVFDGKPQH	487	501	1.22	PP3
16	DRB1*03:01	REGYEMVFDGKPQHT	488	502	1.19	
17	DRB1*03:01	EGYEMVFDGKPQHTN	489	503	1.19	
18	DRB1*03:01	GYEMVFDGKPQHTNV	490	504	1.11	
19	DRB1*03:01	YEMVFDGKPQHTNVC	491	505	1.12	
20	DRB1*03:01	NILLQYVVKSFDRST	116	130	0.98	
21	DRB1*03:01	ILLQYVVKSFDRSTK	117	131	1.09	
22	DRB1*03:01	KVNFFRMVISNPAAT	553	567	1	PP4
23	DRB1*03:01	VNFFRMVISNPAATH	554	568	0.7	
24	DRB1*03:01	NFFRMVISNPAATHQ	555	569	0.69	
25	DRB1*03:01	FFRMVISNPAATHQD	556	570	0.7	
26	DRB1*03:01	FRMVISNPAATHQDI	557	571	0.7	
27	DQA1*05:01‐DQB1*02:01	THQDIDFLIEEIERL	567	581	1.04	
28	DQA1*05:01‐DQB1*02:01	HQDIDFLIEEIERLG	568	582	1.21	
29	DQA1*05:01‐DQB1*02:01	STGLDMVGLAADWLT	183	197	0.95	
30	DQA1*05:01‐DQB1*02:01	TGLDMVGLAADWLTS	184	198	1.28	

Abbreviations: GAD65, glutamic acid decarboxylase 65; PP1, peptide pool 1 (containing peptide 1–7); PP2, peptide pool 2 (containing peptide 8–14); PP3, peptide pool 3 (containing peptide 15–21); PP4, peptide pool 4 (containing peptide 22–30).

### 2.6 T cell stimulation assay

Each of the four GAD65 peptide pools (PP1, 2, 3, and 4) was used to separately stimulate ~5.0 × 10^5^ PBMCs, which were suspended in RPMI media supplemented with 10% FBS (Gibco, USA). The PBMCs along with peptides (final volume: 1 mL) were cultured for 16 h at 37°C with 5% CO_2_ and the culture was supplemented with human interleukin (IL)‐2 (15 units/mL) (Invitrogen, USA), 1 μL of protein transport inhibitor cocktail of brefeldin A and monensin (eBioscience, USA) was added in the beginning of the culture to stop the release of cytokine in the culture. Alongside the four test PPs, a positive control (PMA, phorbol 12‐myristate 13‐acetate/Ionomycin) (eBioscience, USA) and a negative control (without any stimulant/peptide) were also set up. The negative control represented the baseline values of all the cytokines studied. After the completion of the culture, the cells were analyzed on a flow cytometer.

### 2.7 Flow cytometry analysis

The cells were washed with sterile 1xPBS buffer and resuspended in staining buffer (1xPBS with 0.5% sodium azide and 0.5% FBS). A two‐step staining protocol was used to prepare the cells for acquisition using flow cytometer. In the first step, the cell surface molecules were stained with PerCP Mouse Anti‐Human CD3 (552851, BD Biosciences, USA), BB700 Mouse Anti‐Human CD4 (560158, BD Biosciences, USA) and APC‐H7 mouse anti‐human CD8 (560179, BD Biosciences, USA) antibodies. A fixable viability stain 520 (564407, BD Biosciences, USA) was used for assessing the cell viability. Before the second step staining of intracellular cytokines, the cells were permeabilized and fixed using cytoperm/cytofix buffer (554714, BD Biosciences, USA). The intracellular cytokine staining was performed on permeabilized and fixed cells with BV480 mouse anti‐human interferon‐gamma (IFN‐γ; 566100, BD Biosciences, USA), APC mouse anti‐human tumor necrosis factor‐alpha (TNF‐α; 554514, BD Biosciences, USA), PE mouse anti‐human IL‐17A (560486, BD Biosciences, USA), and BV421 rat anti‐human and viral IL‐10 (564053, BD Biosciences, USA) antibodies. Following this, the cells were acquired on BD‐FACSCanto™ II flow cytometer using Diva software (BD Biosciences, USA).

### 2.8 Statistical analysis

For calculating the frequencies of HLA‐DRB1*03‐DQA1*05‐DQB1*02 positive subjects, the number of individuals carrying this haplotype were directly counted and then divided by the total number of individuals, that is, T1D patients or HC. Chi‐square test was used to study the genetic association of HLA‐DRB1*03‐DQA1*05‐DQB1*02 haplotype with T1D and GADA prevalence among the study groups (ie, T1D patients and HC group). Stimulation assay data are represented as the stimulation index (SI), which was calculated using the following formula:

$$\mathrm{SI}=\frac{\%\text{of stimulated}\;\mathrm{CD}4\;T\;\text{cells expressing cytokine}-\%\text{of unstimulated}\;\mathrm{CD}4\;T\;\text{cells expressing cytokine}}{\%\text{of unstimulated}\;\mathrm{CD}4\;T\;\text{cells expressing cytokine}}$$

For analyzing cytokine profiles of CD4 T cells, we used Student's t‐test and the interpeptide group comparisons were performed using one‐way analysis of variance (ANOVA) test. A *p* value of <.05 was determined to be statistically significant. FlowJo software (v10.8.1) was used for analysis of flow cytometry data. For the analysis of HLA, GADA, and flow cytometry data, GraphPad Prism 5.0 Software (San Diego, USA) was used. The odds ratio (OR with 95% onfidence interval [CI]) was calculated and based on this, the risk conferred by the HLA‐DRB1*03‐DQA1*05‐DQB1*02 haplotype was determined.

## 3 RESULTS

### 3.1 Status of GAD65 autoantibody and HLA‐DRB1*03‐DQA1*05‐DQB1*02 haplotype in T1D patients vs healthy controls

Out of 110 T1D patients, GADA was found to be present in 56 (50.91%) patients as compared to that in only 1 out of 127 healthy subjects (0.79%; *p* < .0001; OR = 130.67 [95% CI, 22.18–763.5]; Table 3). Similarly, 87 (79.09%) out of 110 T1D patients carried the HLA‐DRB1*03‐DQA1*05‐DQB1*02 haplotype, which was significantly higher than that in controls wherein only 21 (16.54%) out of 127 carried this haplotype (*p* < .0001; OR = 19.09 [95% CI, 9.94–36.69]; Table 3).

**TABLE 3:** Prevalence of GAD65 autoantibody (GADA) and HLA‐DRB1*03‐DQA1*05‐DQB1*02 haplotype in T1D patients vs healthy controls.

GADA or HLA‐DR3 haplotype status	T1D patients (*N* = 110)	Healthy Contols (*N* = 127)	OR (95% CI)	*p* value
GADA positive	56 (50.91%)	1 (0.79)	130.67 (22.18–763.5)	<.0001
HLA‐DRB1*03‐DQA1*05‐DQB1*02 positive	87 (79.09%)	21 (16.54%)	19.09 (9.94–36.69)	<.0001

Abbreviations: CI, confidence interval; GAD65, glutamic acid decarboxylase; GADA, GAD65 autoantibody; N, number of study subjects; OR, odds ratio; T1D, type 1 diabetes.

The prevalence of GADA in recently diagnosed (RD) T1D patients was slightly higher than that in long‐standing (LS) T1D patients (60.87% vs 48.28%) but this difference was not statistically significant. The prevalence of GADA was also comparable in HLA‐DRB1*03‐DQA1*05‐DQB1*02 positive T1D patients with <2 years disease duration vs ≥2 years disease duration (52.17% vs 41.38%). Further, the prevalence of GADA in patients clinically diagnosed with T1D at >18 years of age and those diagnosed at ≥18 years of age were found to be comparable in this study (55.56% vs 44.68%). Similarly, prevalence of GADA was also comparable in HLA‐DRB1*03‐DQA1*05‐DQB1*02 positive patients diagnosed with T1D at <18 years vs ≥18 (49.21% vs 38.30%). The detailed data are given in the Table S1 in Data S1.

### 3.2 Characterization of GAD65 peptides mediating CD4 T cell response through HLA‐DR3 molecule, HLA‐DQ2 molecule, or both

#### 3.2.1 CD4 T cell response against GAD65 peptides in T1D vs healthy controls

**FIGURE 1:**
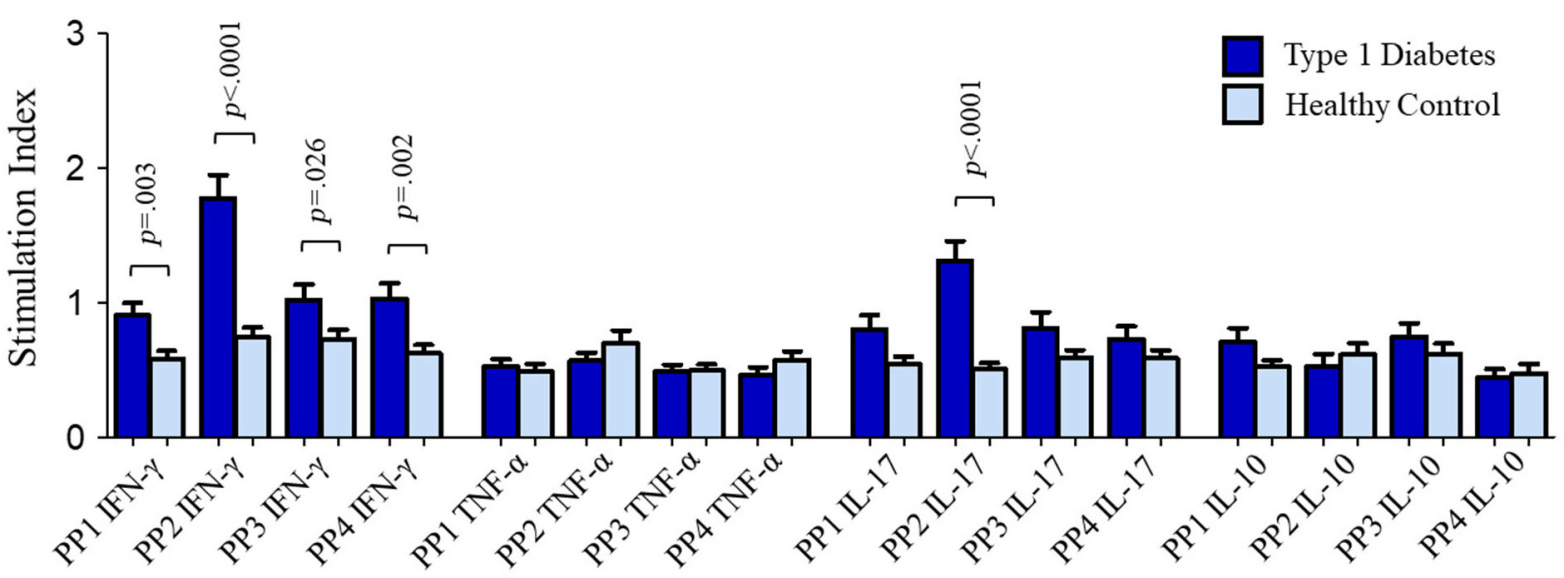
Stimulation indices of CD4 T cells expressing IFN‐γ, TNF‐α, IL‐17, and IL‐10 in response to stimulation with four different GAD65 peptide pools (PP1, PP2, PP3, and PP4) in type 1 diabetes patients and their comparison with that in healthy controls. The results are expressed as mean ± SEM. IFN‐γ, interferon gamma; IL, interleukin; PP, peptide pool; PP1–4 IFN‐γ, PP‐stimulated CD4 T cells expressing IFN‐γ; PP1–4 TNF‐α, PP‐stimulated CD4 T cells expressing TNF‐α; PP1–4 IL‐17, PP‐stimulated CD4 T cells expressing IL‐17; PP1–4 IL‐10, PP‐stimulated CD4 T cells expressing IL‐10; TNF‐α, tumor necrosis factor alpha

When stimulated with all four peptide pools (PPs), the expression of IFN‐γ by CD4 T cells was significantly increased in T1D patients compared to that in HCs (SI‐PP1: 0.91 ± 0.09 in T1D vs 0.58 ± 0.01 in HC, *p* = .003; SI‐PP2: 1.8 ± 0.17 vs 0.74 ± 0.07, *p* < .0001; SI‐PP3: 1.0 ± 0.12 vs 0.73 ± 0.07, *p* = .026 and SI‐PP4: 1.0 ± 0.12 vs 0.62 ± 0.06, *p* = .002) (Figure 1). The stimulation of CD4 T cells with PP2 also resulted in significant increase in the expression of IL‐17 in patients compared to HCs (SI: 1.3 ± 0.15 vs 0.50 ± 0.05, *p* < .0001) (Figure 1). The stimulation indices of TNF‐α and IL‐10 expressing CD4 T cells were found to be comparable between T1D patients and healthy controls when stimulated by the four GAD65 PPs (Figure 1). Representative flow cytometry graphs showing CD4 T cells’ expression of IFN‐γ, TNF‐α, IL‐17, and IL‐10 when cells were stimulated with different stimulants (PMA, PP1, PP2, PP3, and PP4) and unstimulated negative controls (baseline), in T1D patients and HCs, are shown in S2–S5 in Data S1, respectively.

**FIGURE 2:**
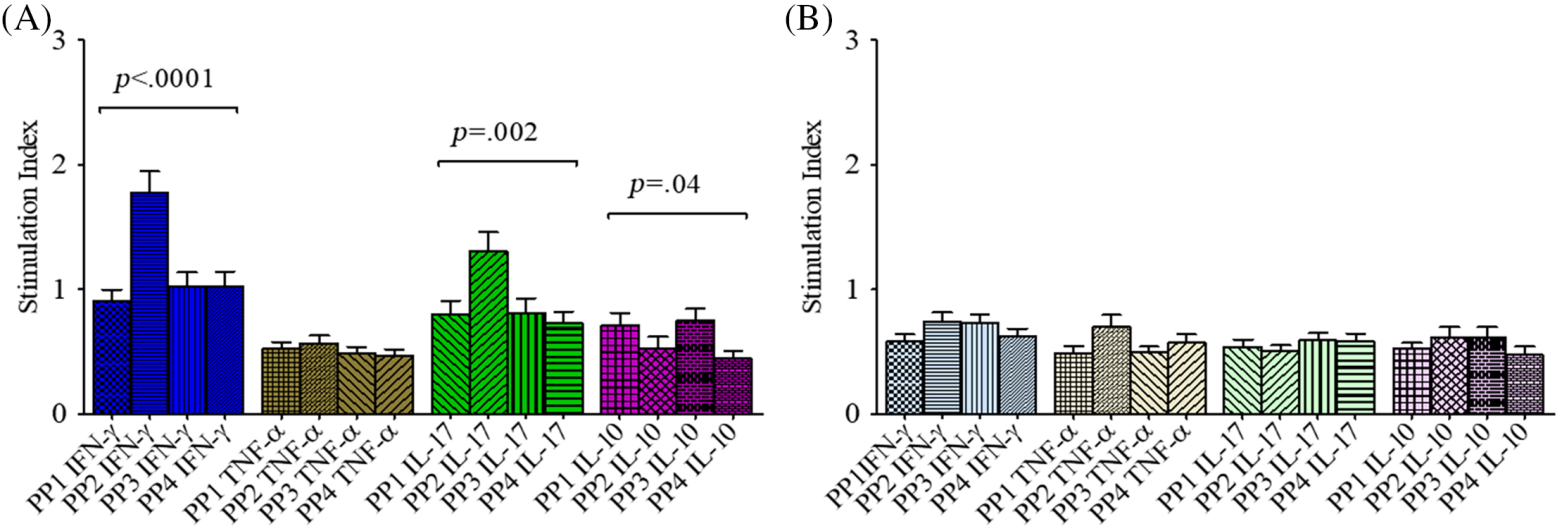
Multiple comparison (one‐way analysis of variance) analysis of stimulation indices of CD4 T cells expressing IFN‐γ (blue), TNF‐α (brown), IL‐17 (green), and IL‐10 (violet) following stimulation with four peptide pools (PP 1–4) in T1D patients (A) and healthy controls (B). The results are expressed as mean ± SEM. IFN‐γ, interferon gamma; IL, interleukin; PP, peptide pool; T1D, type 1 diabetes; TNF‐α, tumor necrosis factor alpha.

Interpeptide group comparison for immunogenicity revealed significant differences in the SIs of IFN‐γ, IL‐17, and IL‐10 expressions by CD4 T cells (*p* < .0001; *p* = .002, and *p* = .04, respectively) in T1D patients only but not in HCs (Figure 2). PP2 was observed to induce highest expression of IFN‐γ and IL‐17 (SI‐PP2: 1.8 ± 0.17 for IFN‐γ and 1.3 ± 0.15 for IL‐17) in T1D patients as compared to that by other pools of peptides (SI‐PP1: 0.91 ± 0.09, SI‐PP3: 1.0 ± 0.12, and SI‐PP4: 1.0 ± 0.12 for IFN‐γ; SI‐PP1: 0.80 ± 0.11, SI‐PP3: 0.81 ± 0.12, and SI‐PP4: 0.73 ± 0.10 for IL‐17). Further, PP2 and PP4 were observed to induce lower expressions of IL‐10 (SI‐PP2: 0.53 ± 0.09 and SI‐PP4: 0.45 ± 0.06) in T1D patients as compared to that by peptide pools 1 and 3 (SI‐PP1: 0.71 ± 0.10 and SI‐PP3: 0.75 ± 0.10).

#### 3.2.2 HLA‐DRB1*03‐DQA1*05‐DQB1*02 mediated CD4 T cell response to GAD65 peptides in T1D patients vs healthy controls

**FIGURE 3:**
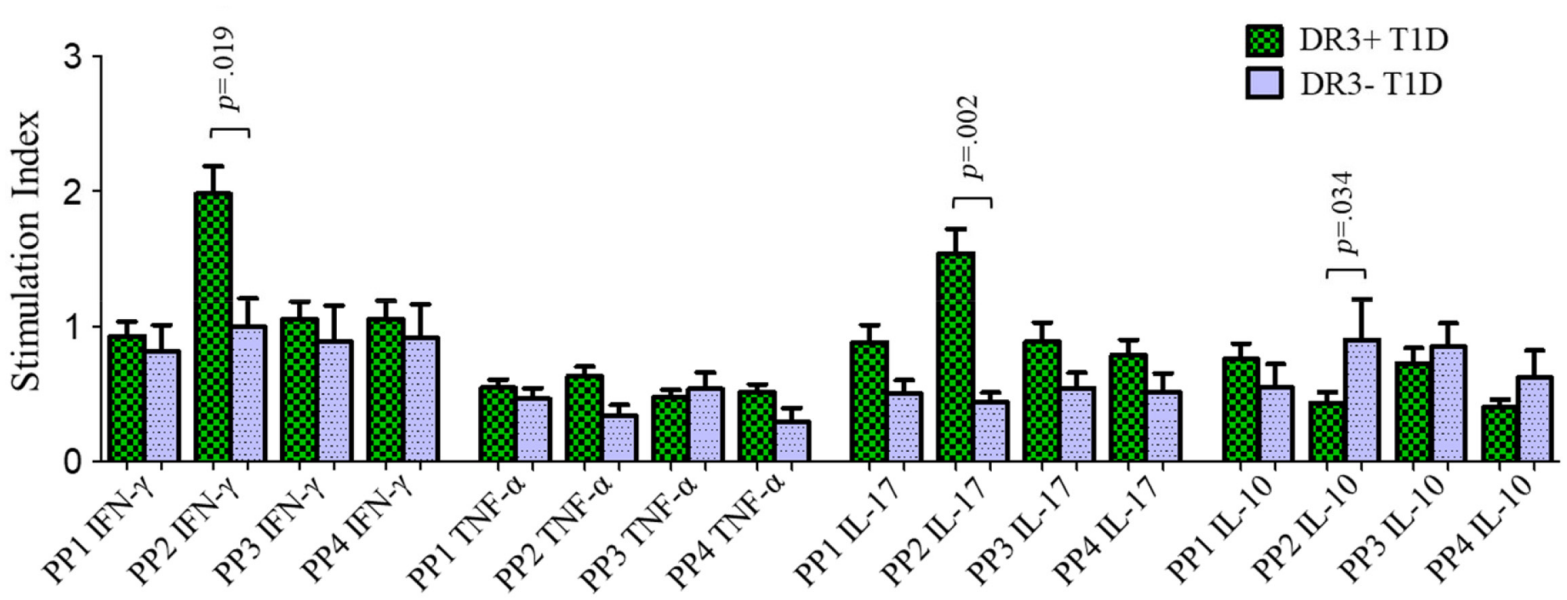
Stimulation indices of CD4 T cells expressing IFN‐γ, TNF‐α, IL‐17, and IL‐10 cytokines in response to stimulation with four different GAD65 peptide pools (PP1, PP2, PP3, and PP4) in HLA‐DRB1*03‐DQA1‐DQB1*02 positive vs negative patients. The results are expressed as mean ± SEM. IFN‐γ, interferon gamma; IL, interleukin; PP, peptide pool; T1D, type 1 diabetes; TNF‐α, tumor necrosis factor alpha.

The culture of PBMCs with PP2 brought about a significantly higher expression of IFN‐γ and IL‐17 cytokines by CD4 T cells in HLA‐DRB1*03‐DQA1*05‐DQB1*02 positive T1D patients compared to those patients not having this haplotype (SI‐PP2: 2.0 ± 0.21 vs. 1.0 ± 0.20, *p* = .019 for IFN‐γ and 1.5 ± 0.18 vs 0.44 ± 0.07, *p* = .002 for IL‐17) (Figure 3). The expression of TNF‐α by the CD4 T cells was found to be comparable between HLA‐DRB1*03‐DQA1*05‐DQB1*02 positive and negative T1D patients. Further, PP2 stimulation caused a significant decrease in the CD4 T cells' expression of IL‐10 in HLA‐DRB1*03‐DQA1*05‐DQB1*02 positive vs negative T1D patients (0.43 ± 0.08 vs 0.90 ± 0.30, *p* = .034) (Figure 3). Representative flow cytometric graphs showing CD4 T cells' expression of IFN‐γ, TNF‐α, IL‐17, and IL‐10 when cells were stimulated with different stimulants (PMA, PP1, PP2, PP3, and PP4) and unstimulated negative controls (baseline) in HLA‐DRB1*03‐DQA1*05‐DQB1*02 positive and negative T1D patients as well as HLA‐DRB1*03‐DQA1*05‐DQB1*02 positive and negative HCs are shown in Figures S6–S9 in Data S1.

**FIGURE 4:**
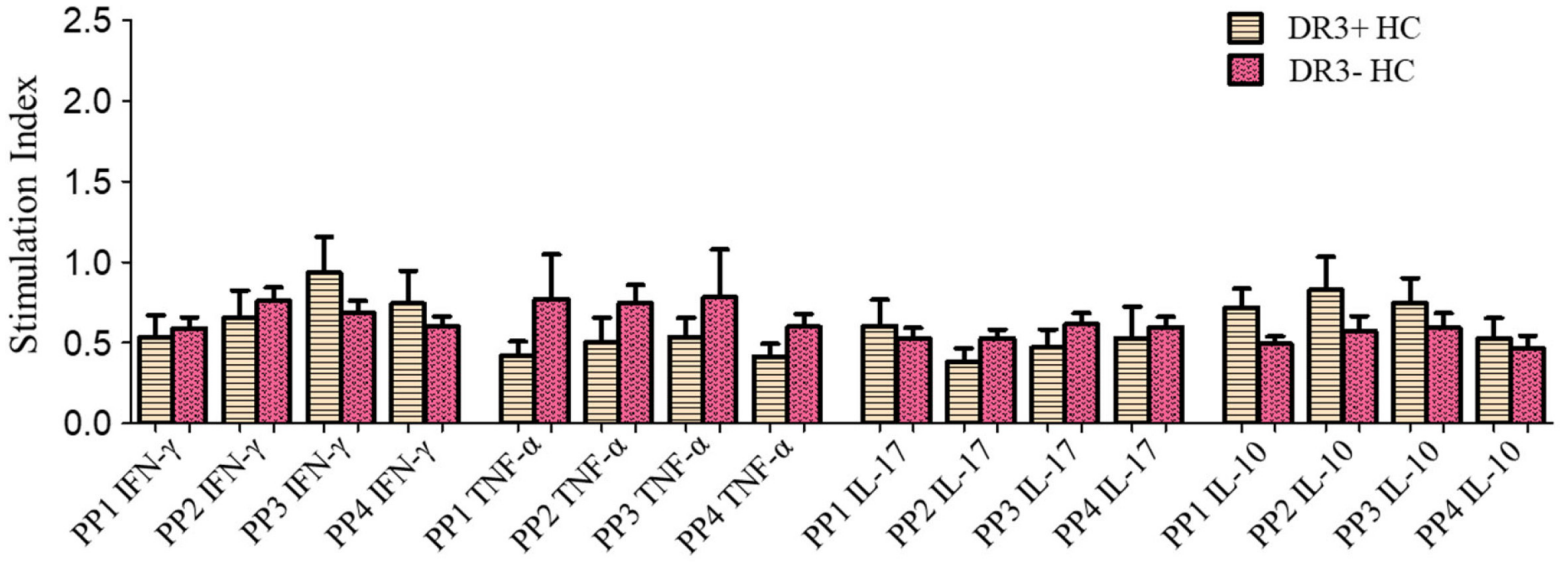
Stimulation indices of CD4 T cells expressing IFN‐γ, TNF‐α, IL‐17, and IL‐10 cytokines in response to stimulation with four different GAD65 peptide pools (PP1, PP2, PP3, and PP4) in HLA‐DRB1*03‐DQA1*05‐DQB1*02 positive vs negative healthy controls. The results are expressed as mean ± SEM. HC, healthy controls; IFN‐γ, interferon gamma; IL, interleukin; PP, peptide pool; TNF‐α, tumor necrosis factor alpha.

The stimulation of PBMCs with all four PPs showed no significant difference in the CD4 T cells' expression of any of IFN‐γ, TNF‐α, IL‐1,7 and IL‐10 cytokines in HC carrying HLA‐DRB1*03‐DQA1*05‐DQB1*02 haplotype versus those who do not carry this haplotype (Figure 4).

**FIGURE 5:**
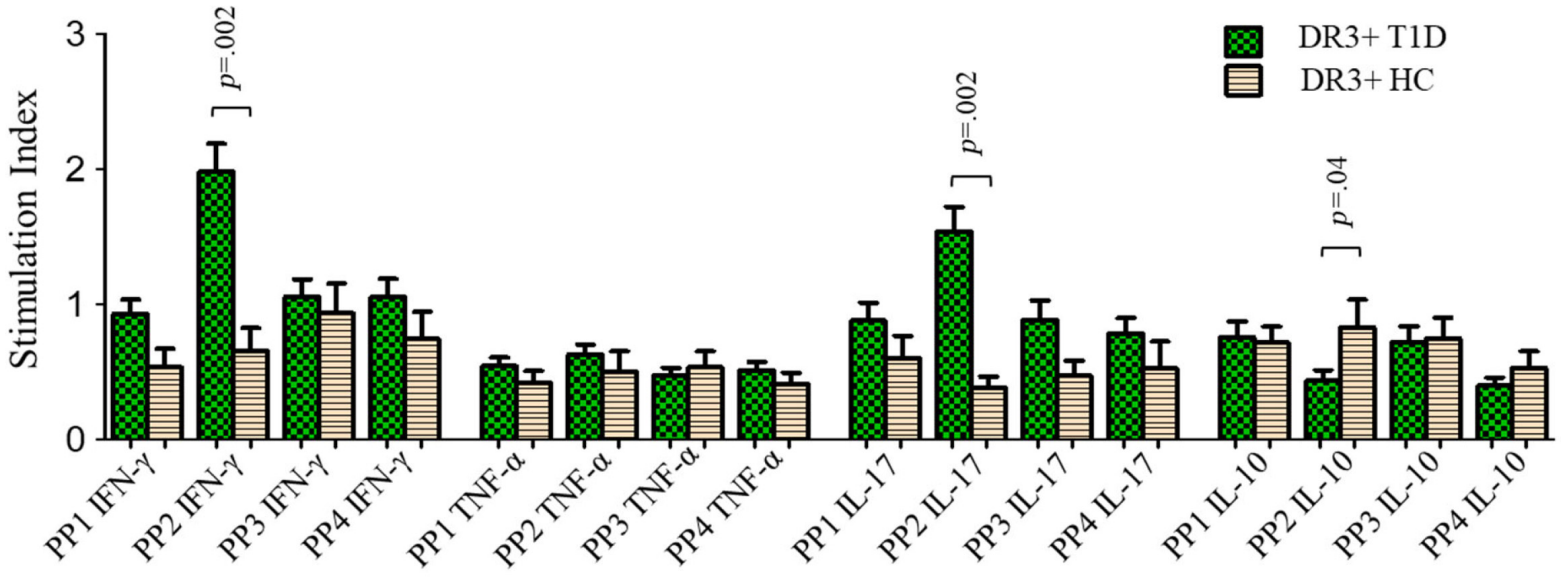
Stimulation indices of CD4 T cells expressing IFN‐γ, TNF‐α, IL‐17, and IL‐10 cytokines in response to stimulation with four different GAD65 peptide pools (PP1, PP2, PP3, and PP4) in HLA‐DRB1*03‐DQA1*05‐DQB1*02 positive T1D patients and HLA‐DRB1*03‐DQA1*05‐DQB1*02 positive healthy controls. The results are expressed as mean ± SEM. HC, healthy controls; IFN‐γ, interferon gamma; IL, interleukin; PP, peptide pool; SEM, standard error of mean; T1D, type 1 diabetes; TNF‐α, tumor necrosis factor alpha.

Compared with those in HLA‐DRB1*03‐DQA1*05‐DQB1*02 positive HC, the SIs of IFN‐γ and IL‐17 expressing CD4 T cells were found to be significantly increased in HLA‐DRB1*03‐DQA1*05‐DQB1*02 positive T1D patients (2.0 ± 0.21 in T1D vs 0.66 ± 0.17 in HCs, *p* = .002 for IFN‐γ and 1.5 ± 0.18 vs 0.38 ± 0.08, *p* = .002 for IL‐17) (Figure 5). On the other hand, the expression of IL‐10 by CD4 T cells was found to be significantly reduced in HLA‐DRB1*03‐DQA1*05‐DQB1*02 positive T1D patients compared to that in HLA‐DRB1*03‐DQA1*05‐DQB1*02 positive HC (0.43 ± 0.08 vs 0.83 ± 0.20, *p* = .04) (Figure 5). The expression of TNF‐α by CD4 T cells was found to be comparable between HLA‐DRB1*03‐DQA1*05‐DQB1*02 positive T1D patients and HLA‐DRB1*03‐DQA1*05‐DQB1*02 positive HC (Figure 5).

**FIGURE 6:**
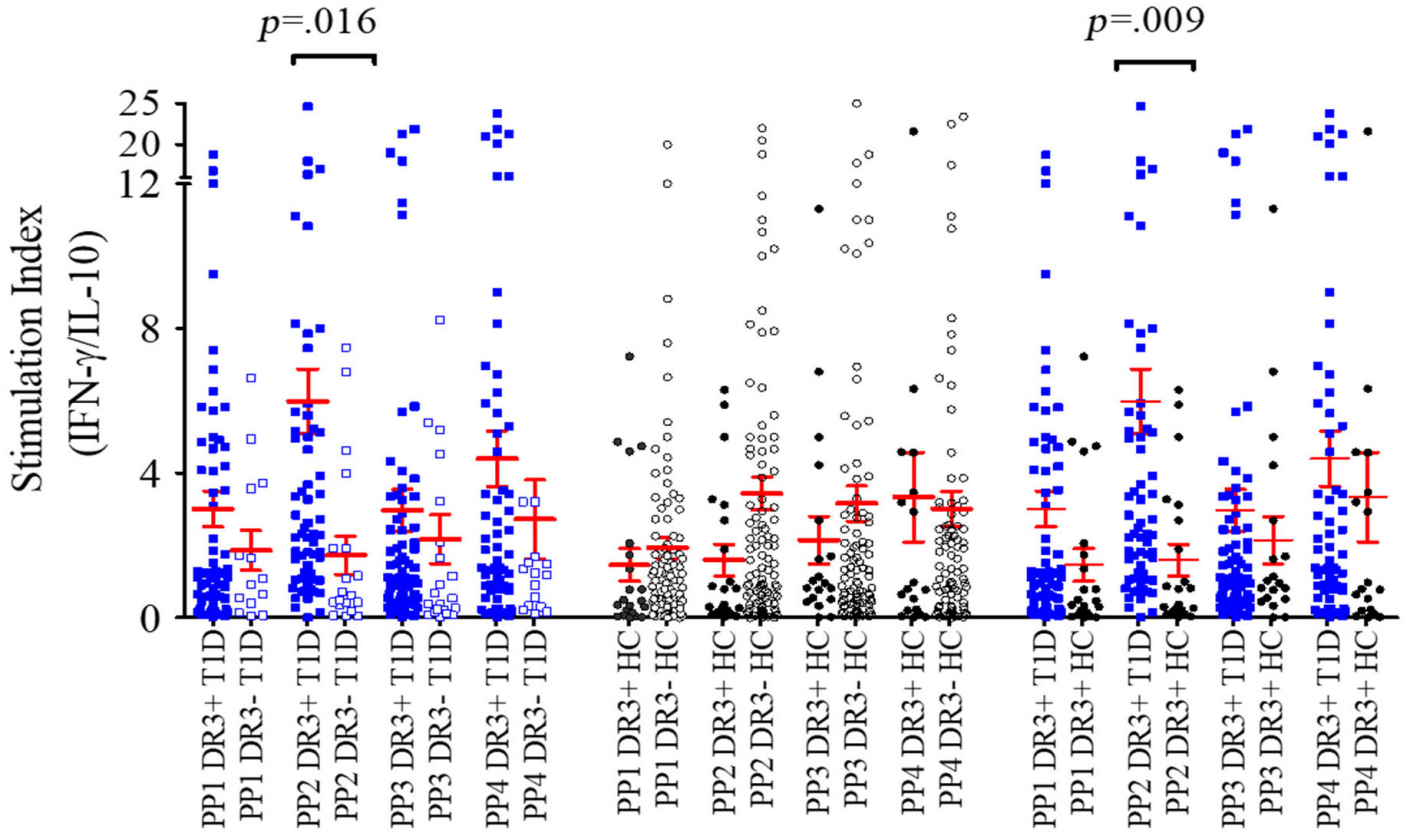
Ratios of stimulation indices for IFN‐γ/IL‐10 when CD4 T cells were stimulated with four different GAD65 peptide pools (PP1, PP2, PP3, and PP4) in HLA‐DRB1*03‐DQA1*05‐DQB1*02 positive vs negative T1D patients, HLA‐DRB1*03‐DQA1*05‐DQB1*02 positive vs negative healthy controls and HLA‐DRB1*03‐DQA1*05‐DQB1*02 positive T1D vs HLA‐DRB1*03‐DQA1*05‐DQB1*02 positive healthy controls. The results are expressed as mean ± SEM. HC, healthy controls; IFN‐γ, interferon gamma; IL, interleukin; PP, peptide pool; SEM, standard error of mean; T1D, type 1 diabetes; TNF‐α, tumor necrosis factor alpha.

**FIGURE 7:**
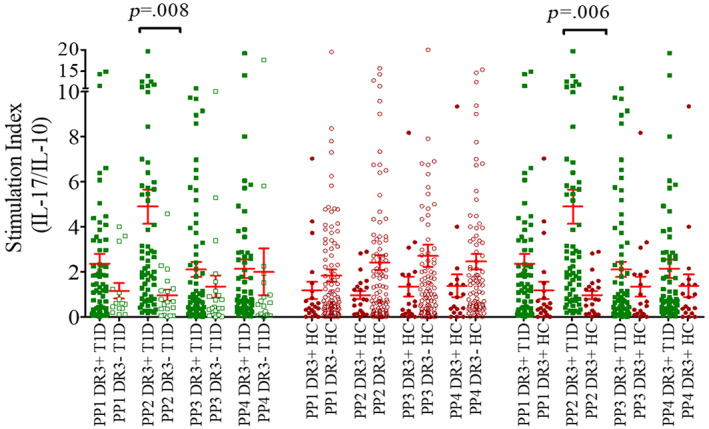
Ratios of stimulation indices for IL‐17/IL‐10 when the CD4 T cells were stimulated with four different GAD65 peptide pools (PP1, PP2, PP3, and PP4) in HLA‐DRB1*03‐DQA1*05‐DQB1*02 positive vs negative T1D patients, HLA‐DRB1*03‐DQA1*05‐DQB1*02 positive vs negative healthy controls and HLA‐DRB1*03‐DQA1*05‐DQB1*02 positive T1D vs HLA‐DRB1*03‐DQA1*05‐DQB1*02 positive healthy controls. The results are expressed as mean ± SEM. HC, healthy controls; IFN‐γ, interferon gamma; IL, interleukin; PP, peptide pool; SEM, standard error of mean; T1D, type 1 diabetes; TNF‐α, tumor necrosis factor alpha.

When compared with HLA‐DRB1*03‐DQA1*05‐DQB1*02 negative T1D group, the ratios of SI for IFN‐γ/IL‐10 and IL‐17/IL‐10 cytokine expressions by CD4 T cells, in response to PP2 of GAD65, were significantly increased in HLA‐DRB1*03‐DQA1*05‐DQB1*02 positive patients (ratio: 6.0 ± 0.90 in DR3+ T1D vs. 1.7 ± 0.53 in DR3– T1D, *p* = .016 for IFN‐γ/IL‐10 and 4.9 ± 0.76 vs 0.95 ± 0.26, *p* = .008 for IL‐17/IL‐10) (Figures 6 and 7). Similarly, when compared with HLA‐DRB1*03‐DQA1*05‐DQB1*02 positive HC, the SI ratios of IFN‐γ/IL‐10 and IL‐17/IL‐10 cytokines were significantly elevated in HLA‐DRB1*03‐DQA1*05‐DQB1*02 positive T1D patients (6.0 ± 0.9 in DR3+ T1D vs 1.6 ± 0.44 in DR3+ HC, *p* = .009 for IFN‐γ/IL‐10 and 4.9 ± 0.76 vs 0.96 ± 0.19, *p* = .006 for IL‐17/IL‐10) (Figures 6 and 7). However, when stimulated with other three GAD65 PPs (ie, PP1, PP3, and PP4), the IFN‐γ/IL‐10 and IL‐17/IL‐10 ratios were comparable between patients and controls carrying this haplotype. Further, the ratios of SI for IFN‐γ/IL‐10 and IL‐17/IL‐10 cytokine expressions by CD4 T cells, in response to the four PPs of GAD65, were found to be comparable in HLA‐DRB1*03‐DQA1*05‐DQB1*02 positive and negative HC (Figures 6 and 7).

#### 3.2.3 CD4 T cell response to GAD65 peptides in T1D patients grouped on the basis of disease duration and age at T1D onset

**FIGURE 8:**
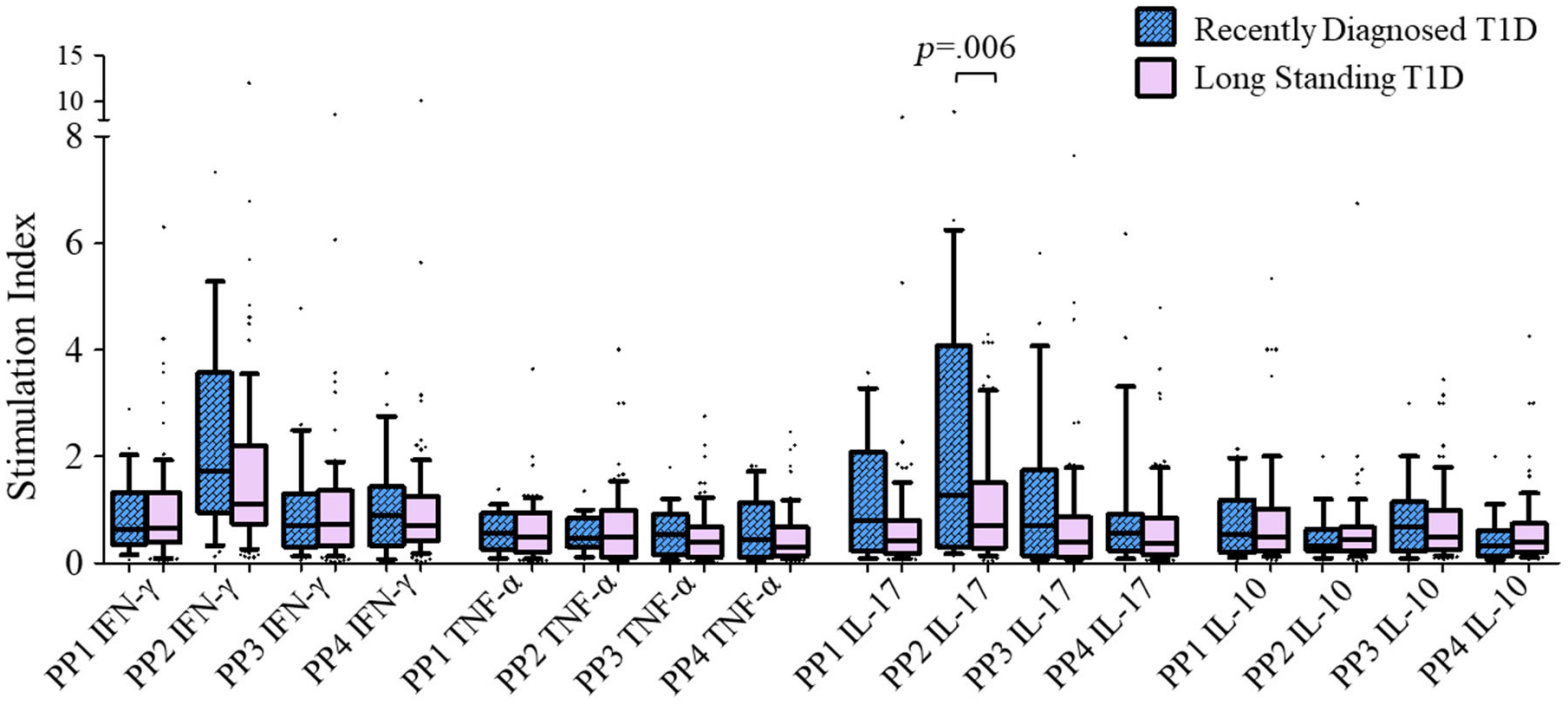
Stimulation indices of CD4 T cells' expression of IFN‐γ, TNF‐α, IL‐17, and IL‐10 when the cells were stimulated with four different GAD65 peptide pools (PP1, PP2, PP3, and PP4) in recently diagnosed and long‐standing T1D patients. The results are represented by box and whisker plots where boxes represent mean ± SEM and whiskers represent 10–90 percentile data range. IFN‐γ, interferon gamma; IL, interleukin; PP, peptide pool; T1D, type 1 diabetes; TNF‐α, tumor necrosis factor alpha.

**FIGURE 9:**
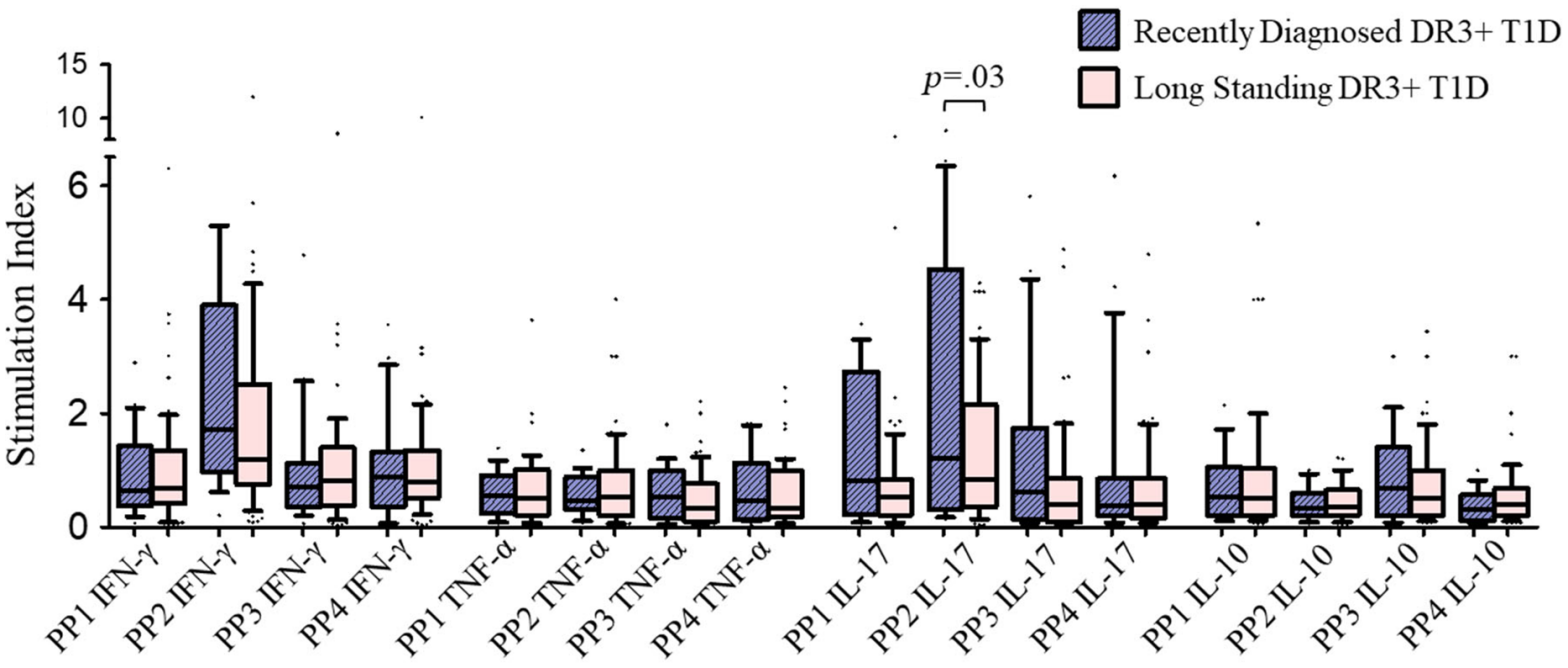
Stimulation indices of CD4 T cells' expression of IFN‐γ, TNF‐α, IL‐17, and IL‐10 when the cells were stimulated with four different GAD65 peptide pools (PP1, PP2, PP3, and PP4) in recently diagnosed and long‐standing HLA‐DRB1*03‐DQA1*05‐DQB1*02 positive T1D patients. The results are represented by box and whisker plots where boxes represent mean ± SEM and whiskers represent 10–90 percentile data range. IFN‐γ, interferon gamma; IL, interleukin; PP, peptide pool; T1D, type 1 diabetes; TNF‐α, tumor necrosis factor alpha.

On the basis of disease duration, we further divided T1D patients into two groups: (a) RD patients with disease duration of <2 years and (b) LS T1D patients with disease duration of ≥2 years. Our data revealed that the GAD65 PP2 stimulation resulted in the significant increase in the expression of IL‐17 cytokine by CD4 T cells in RD patients compared to those with LS T1D patients (2.1 ± 0.53 vs 1.1 ± 0.12, *p* = .006) (Figure 8). The stimulation with PP1, PP3, and PP4 resulted in comparable expression of IL‐17 in RD vs LS patients. Similarly, when stimulated with all four GAD65 PPs, the expression of IFN‐γ, TNF‐α, and IL‐10 by CD4 T cells was comparable between RD and LS T1D patients (Figure 8). Further, GAD65 PP2 stimulation resulted in significantly higher expression of IL‐17 cytokine in HLA‐DRB1*03‐DQA1*05‐DQB1*02 positive RD T1D patients compared to that in LS patients carrying this haplotype (2.2 ± 0.57 vs. 1.3 ± 0.15, *p* = .03) (Figure 9).

The analysis on the basis of age at disease onset showed that the stimulation by all four GAD65 PPs resulted in similar expression of IFN‐γ, TNF‐α, IL‐17, and IL‐10 cytokines by CD4 T cells in patients with age at onset <18 versus ≥18 years. Similarly, GAD65 PP stimulation resulted in comparable expressions of the four cytokines in HLA‐DRB1*03‐DQA1*05‐DQB1*02 positive patients diagnosed with T1D at age <18 years vs ≥18 years (data shown in Figures S10, S11 in Data S1). Further, similar results were also obtained for HCs stratified based on their age at the time of the study that is, age <18 years vs ≥18 years (Figure S12 in Data S1).

#### 3.2.4 CD4 T cell response to GAD65 in patients with and without GADA

The comparison between patients with GADA and without GADA for CD4 T cell response against all four GAD65 PPs revealed that SIs for expression of IFN‐γ, TNF‐α, IL‐17, and IL‐10 by CD4 T cells were comparable between GADA positive and negative patients (Figure S13 in Data S1). We further evaluated the GADA titer vs expression level of each cytokine (ie, IFN‐γ, TNF‐α, IL‐17, and IL‐10) production upon stimulation with the four GAD65 PPs at the individual level. Our data did not show any correlation between GADA titers and expression of cytokines by CD4 T cells in T1D patients (data shown in Figure S14A–D in Data S1).

## 4 DISCUSSION

The phenomenon of epitope‐specific immune response is well documented in chronic T cell‐mediated autoimmune disorders including T1D. Results from animal models as well as human studies on T1D have demonstrated that a major event in the disease process is the presentation of diabetogenic peptides by susceptible HLA class II molecule to CD4 T cells.^5^, ^11^, ^24^, ^25^ Genetically, the most significant risk factor associated with T1D in North India is the presence of the HLA‐DRB1*03 gene and its associated HLA‐DRB1*03‐DQA1*05‐DQB1*02 haplotype.^15^, ^16^, ^17^ Further, HLA‐DRB1*03‐DQA1*05‐DQB1*02 haplotype is reported to be strongly associated with the presence of GADA in T1D patients.^19^, ^20^, ^26^, ^27^, ^28^ A recent study that investigated the association between epitope‐specific GADA and the HLA‐DR‐DQ haplotypes revealed that T1D‐susceptible HLA haplotypes, particularly DR3 positive haplotypes, associate strongly with GAD65Ab multiepitope positivity, suggesting that an important pathological mechanism in T1D might involve the presentation of GAD65 peptides by both HLA‐DR and HLA‐DQ molecules.^29^ The present study was carried out to characterize the GAD65 epitopes possibly presented by HLA‐DR3/DQ2 molecules to CD4 T cells and compare them between T1D patients and HC. We used four pools of GAD65 peptides (the top 30 overlapping peptides, predicted to bind in silico to HLA‐DR3 molecule, HLA‐DQ2 molecule, or both) to stimulate the CD4 T cells for the expression of proinflammatory cytokines IFN‐γ, IL‐17, and TNF‐α and anti‐inflammatory cytokine IL‐10. As each person may have many confounding factors that may elevate or suppress given cytokine production, we represented stimulation assay data as Stimulation Index (SI). Our data revealed that of the four GAD65 PPs, PP2, having seven GAD65 peptides from amino acid 288 to 308 (GAD288‐308), was the most immunogenic and induced significantly higher expressions of IFN‐γ and IL‐17 cytokines by CD4 T cells of T1D patients compared to that of HC. This was further confirmed by interpeptide comparison using one‐way ANOVA, where PP2 was observed to be the predominant pool stimulating the CD4 T cells for the expression of IFN‐γ and IL‐17; however, this preference of PP2 was not observed in HC, suggesting their role in the T1D development.

When we looked at the function of CD4 T cells in response to the stimulation by GAD65 PPs in study subjects carrying HLA‐DRB1*03‐DQA1*05‐DQB1*02 haplotype and compared it to those who were negative for this haplotype, we found that only PP2 resulted in significantly elevated expressions of IFN‐γ and IL‐17 by CD4 T cells in patients carrying this haplotype vs HLA‐DRB1*03‐DQA1*05‐DQB1*02 negative patients but similar results were not observed in HC. The data suggest that PP2 (GAD288‐308) possibly contains the peptides that harbor the epitopes for HLA‐DR3‐restricted autoreactive CD4 T cells. In other words, the data from our study indicate that the CD4 T cells of T1D patients particularly having HLA‐DRB1*03‐DQA1*05‐DQB1*02 haplotype are reactive against a particular region of the GAD65 protein.

When the expression of cytokines by CD4 T cells of HLA‐DRB1*03‐DQA1*05‐DQB1*02 positive T1D patients and HLA‐DRB1*03‐DQA1*05‐DQB1*02 positive HC were compared, our study revealed that PP2 stimulation resulted in a significant increase in the expressions of IFN‐γ and IL‐17 by CD4 T cells and a significant decrease in IL‐10 expression in patients vs controls. These findings suggest the occurrence of GAD65‐reactive CD4 T cells in T1D patients, particularly those carrying HLA‐DRB1*03‐DQA1*05‐DQB1*02 haplotype and not in the healthy controls. Further, we observed a significant increase in IFN‐γ/IL‐10 and IL‐17/IL‐10 ratios in HLA‐DRB1*03‐DQA1*05‐DQB1*02 positive vs negative T1D patients as well as in HLA‐DRB1*03‐DQA1*05‐DQB1*02 positive patients vs healthy controls. Our data indicate that GAD65 PP2 possibly mediated a shift of immune balance toward inflammatory phenotype in T1D only, whereas in HC, the immune balance is maintained. Multiple peptides of GAD65 have been reported to suppress T1D by controlling inflammatory T cells and induction of regulatory T cells.^13^, ^30^, ^31^ Further, the capacity of different epitopes to induce regulatory T cells was reported to be dependent on the frequency of clonotype T cells and the presence of pathogenic GAD65‐reactive T cells, possibly leading to the development of T1D.^31^, ^32^, ^33^

Earlier studies have shown that the magnitude of immune response to antigens can vary with time.^34^, ^35^ We found the prevalence of GADA in our study to be 50.91% in T1D patients compared to just 0.79% in HC (only 1 GADA+ HC out of 127). We could not follow up and confirm whether the HC who showed GADA positive status went on to develop T1D. The median disease duration of T1D patients in this study is 6 years, which is quite long and because GADA in sera is known to decrease with time, this could explain the lower prevalence of GADA in T1D patients in our population compared to other populations that have reported higher GADA prevalence.^18^, ^36^, ^37^, ^38^ It has been reported that GADA positivity is associated with an older age at T1D diagnosis and further GADA‐initiated autoimmunity was associated with longer disease duration.^39^, ^40^ When we grouped our patients on the basis of age at T1D onset and disease duration, we found that prevalence of GADA was comparable in RD (disease duration <2 years) vs LS (≥2 years) T1D patients as well as in patients with age at onset <18 years vs ≥18 years. Our study also revealed that the CD4 T cells' responses to GAD65 peptide stimulation in terms of expression of IFN‐γ, TNF‐α, IL‐17, and IL‐10 cytokines were found to be comparable between patients who were presented with T1D before 18 years of age and those who were diagnosed at or after 18 years of age. These findings possibly indicate that humoral and cellular autoimmunity in T1D patients may be long lasting. The CD4 T cells' response to GAD65 peptides was also observed to be not affected by the age of HC, suggesting that age should not be a confounding factor in this study. Further, our data revealed that GAD65 PP2 induced significantly higher expression of IL‐17 cytokine by CD4 T cells in RD vs LS T1D patients as well as in HLA‐DRB1*03‐DQA1*05‐DQB1*02+ RD vs HLA‐DRB1*03‐DQA1*05‐DQB1*02+ LS T1D patients. These findings suggest a possible important role of IL‐17 producing CD4 T cells (Th17) in driving inflammatory CD4 T cell response in T1D, a role that has been reported in several other studies.^41^, ^42^, ^43^ Surprisingly, when GAD65 peptide‐mediated CD4 T cell stimulation was compared between GADA positive and negative T1D patients, CD4 T cell response was similar as evidenced by the absence of significant difference for any of the cytokines we studied. Further, when we evaluated the correlation of GADA titers with the expression level of each cytokine upon peptide stimulation at the individual level, the CD4 T cells' response to peptide stimulation did not correlate with GAD65 autoantibody titers in patients, suggesting that there may not be a particular preference for antibody titers vs cytokine expression in T1D in this population, which may be because of differences in T and B cell epitopes. Although the current study defined the GAD65 peptides possibly presented by HLA‐DR3 molecule, HLA‐DQ2 molecule, or both to CD4 T cells (CD4 T cell epitopes), a different study may be needed to identify the B cell epitopes.

There are several studies implicating the major role played by proinflammatory cytokines such as IFN‐γ, TNF‐α, and IL‐17 in destruction of pancreatic β‐cells leading to T1D in both animal models and humans.^43^, ^44^, ^45^, ^46^, ^47^, ^48^, ^49^ The production of these cytokines promotes the differentiation of immune cells into autoreactive cells, which attack β‐cells leading to the T1D onset. In this study, we showed that two cytokines, IFN‐γ and IL‐17, might have an important role in GAD65‐specific CD4 T cell immune response against islet β‐cells in T1D patients particularly having HLA‐DRB1*03‐DQA1*05‐DQB1*02 haplotype. However, we found that when stimulated with all four GAD65 PPs, the CD4 T cells' expression of TNF‐α was comparable between T1D patients and controls and between HLA‐DRB1*03‐DQA1*05‐DQB1*02 positive and negative patients, suggesting a minimal or non‐significant role of TNF‐α in the HLA‐DR3/DQ2‐mediated CD4 T cell immune response against the GAD65 protein, which is a prerequisite for T1D development.

In this study, we used an online epitope prediction tool that predicted the top 30 GAD65 peptides that strongly bind in silico to HLA‐DR3 moleculs, HLA‐DQ2 molecule, or both. There are other epitope prediction tools available online probably with different score/rank for these peptides but regardless of the scores obtained from different databases, strong in silico binding does not automatically mean autoimmune response, nor weak binding the opposite. This was also supported by our study in which not all the GAD65 peptides predicted to bind in silico to either HLA‐DR3 or HLA‐DQ2 stimulated CD4 T cells to express proinflammatory cytokines, suggesting that either some peptides that were observed to bind strongly in silico to HLA‐DR3 or DQ2 may not bind in vitro or the CD4 T cells may not be reactive against these peptides. As we have used the top 30 GAD65 peptides from only one database, it is possible that there may be other GAD65 peptides that strongly bind the DR3 molecule, DQ2 molecule, or both in vitro. Nevertheless, we were able to show in this study that GAD288‐308, belonging to group 2 of GAD65 peptides, may harbor epitopes for diabetogenic CD4 T cells in T1D patients carrying HLA‐DRB1*03‐DQA1*05‐DQB1*02 haplotype, suggesting their possible role in T1D pathogenesis. Further, out of the four GAD65 PPs, PP1, PP2, and PP3 were found to bind strongly in silico to the HLA‐DR3 molecule and PP4 consisted of peptides that showed binding affinity to HLA‐DR3 and HLA‐DQ2 molecules. However, only PP2 was found to induce significant inflammatory CD4 T cell response, suggesting an important role of the HLA‐DR3 molecule in CD4 T cell response against GAD65 in T1D. As the processes involved in onset of autoimmunity and progression to T1D are dynamic and complex, and several combinations of events may alter the presentation of self‐peptides to autoreactive T cells,^5^, ^50^, ^51^ further functional studies are needed to better understand the events that lead to the activation of autoreactive CD4 T cells, in response to antigenic peptide presentation by high‐risk HLA‐DR3 molecule, HLA‐DQ2 molecule, or both.

One of the shortcomings of this study is that we used PPs and not individual peptides to stimulate the cells and hence, whether it is the combination of the peptides or one single peptide in PP2 that is responsible for increased expression of IFN‐γ and IL‐17 by CD4 T cells cannot be determined. In this study, we have taken the samples of only clinically diagnosed T1D patients and have not included prediabetic samples and therefore, our data are representative of only clinically diagnosed T1D patients, who were already taking insulin for diabetes management. There is a requirement of functional studies to further validate our findings, which showed that HLA‐DR3 molecule possibly plays a key role in initiating the autoimmune response against GAD65 protein in T1D. Identifying and characterizing the type of immunogenic peptides presented by high‐risk HLA class II molecules to CD4 T cells and cytokines involved in the disease process might be helpful in developing targeted vaccine designing strategies and development of new therapeutics for T1D.

## 5 CONCLUSION

In this research work, we defined GAD65 peptides possibly presented by HLA‐DR3 molecule, HLA‐DQ2 molecule, or both to the CD4 T cells and investigated the DR3/DQ2‐mediated GAD65‐specific CD4 T cells immune response in T1D patients carrying HLA‐DRB1*03‐DQA1*05‐DQB1*02 haplotype. Our study supports the earlier studies that reported that HLA‐DR3 molecule, HLA‐DQ2 molecule, or both are quite selective and present only a few GAD65 peptides to CD4 T cells, which possibly leads to immune response against GAD65 protein in T1D. Our study revealed that the GAD65 peptides encompassing 288–308 amino acid positions are possibly favored by the HLA‐DR3 molecule for presentation to CD4 T cells in T1D. Furthermore, this study showed that two cytokines, namely, IFN‐γ and IL‐17, might have central roles in the T1D development. Functional studies that may be built on our present work and validate our findings are likely to yield valuable information that can contribute to better understanding of the mechanisms involved in T1D development. The findings of this study might be helpful in developing targeted vaccine‐based approaches for prevention of T1D.

## AUTHOR CONTRIBUTIONS

Neihenuo Chuzho performed the experiments, analyzed the data and contributed to writing of the manuscript, Nikhil Tandon provided the study materials and reviewed the manuscript; Uma Kanga, Akanksha Sharma, and Gunja Mishra performed the flow cytometry experiments and data analysis, Neetu Mishra and Narinder K. Mehra gave further insight on result interpretation and reviewed the manuscript, and Neeraj Kumar was involved in conception, supervision, and overall execution of this study and co‐wrote the manuscript. All authors read and approved the final version of the manuscript.

## CONFLICT OF INTEREST STATEMENT

The authors have no conflict of interest to declare.

## Supporting information


**Data S1.** Supporting information.
