# Supplementary material for: HLA‐DR3 mediated CD4 T cell response against GAD65 in type 1 diabetes patients
Source: J Diabetes. 2023 Jun 13;15(7):607–21. doi: 10.1111/1753-0407.13406 (PMC10345980; doi:10.1111/1753-0407.13406)
Supplement: Supplementary file 1 — Data S1. Supporting information. [file JDB-15-607-s001.docx]

**Supplementary Material**


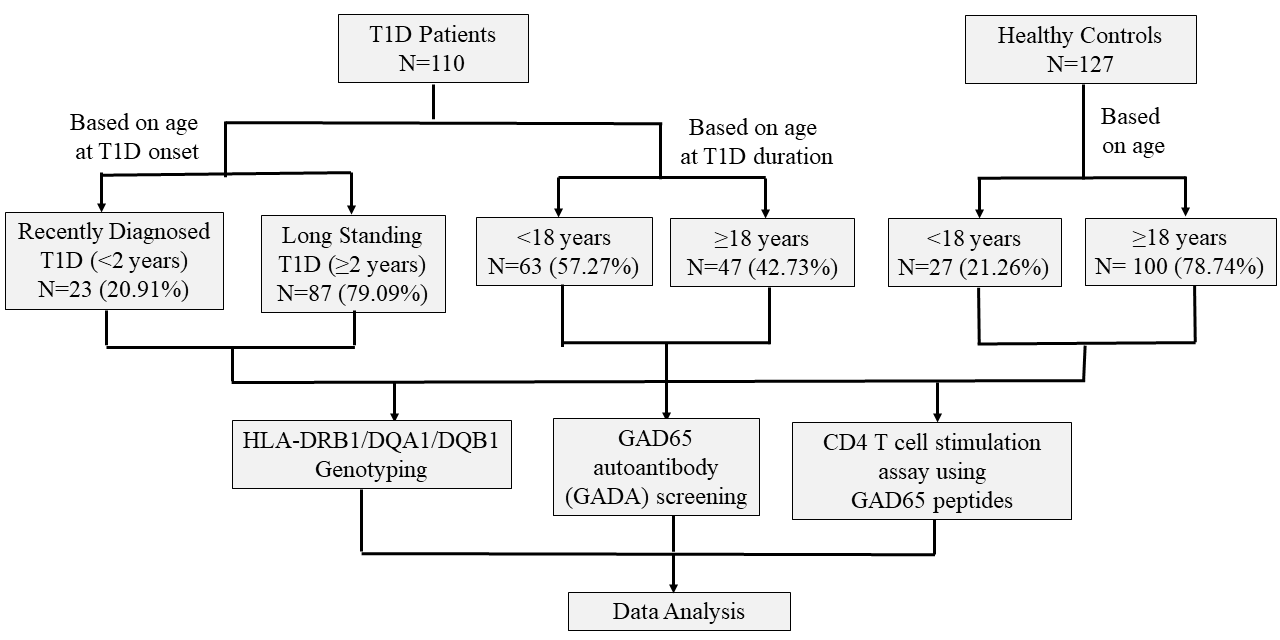
**Supplementary Figure 1**: Workflow of the study showing T1D patients categorised on the basis of age at onset and disease duration and healthy controls categorised on the basis of age at sampling. Abbreviations: N, number of study subjects; T1D, type 1 diabetes.


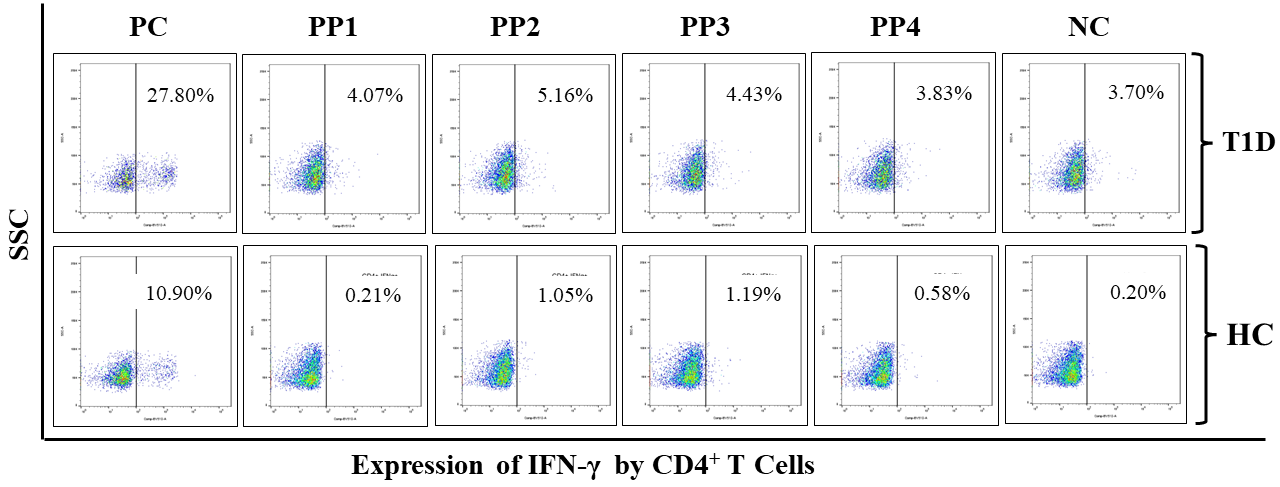
**Supplementary Figure 2**: Representative flow cytometric graphs showing CD4 T cells’ expression of IFN-γ when cells were stimulated with four different peptide pools (PP1, PP2, PP3 and PP4), phorbol 12-myristate 13-acetate (PMA)/ionomycin stimulation (PC), and unstimulated negative controls (NC or baseline) in T1D and healthy controls. Abbreviations: PP, peptide pool; PC, positive control; NC, negative control; SSC, side scatter; T1D, type 1 diabetes; HC, healthy control.


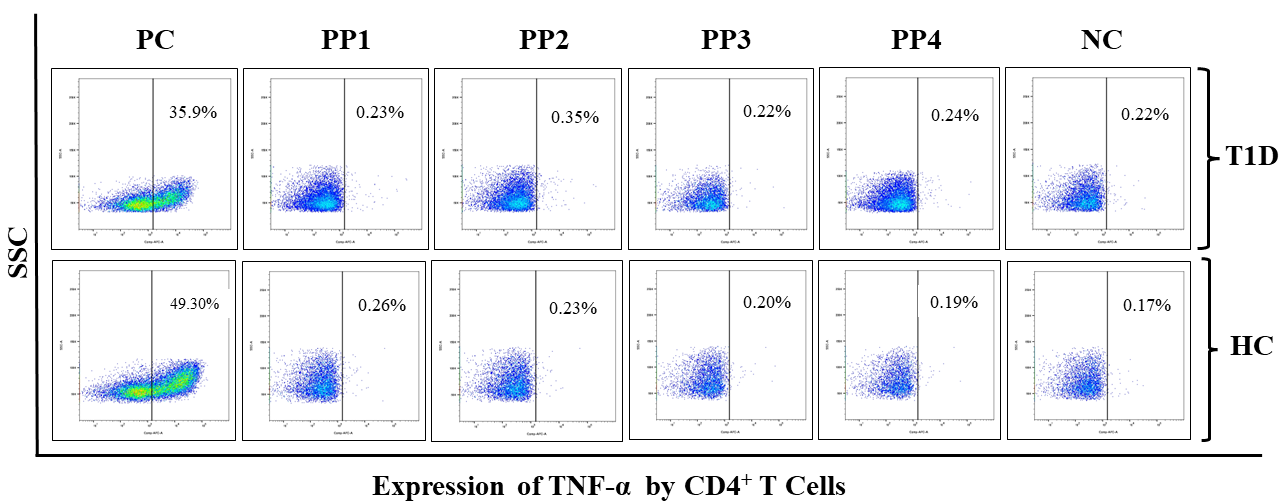
**Supplementary Figure 3**: Representative flow cytometric graphs showing CD4 T cells’ expression of TNF-α when cells were stimulated with four different peptide pools (PP1, PP2, PP3 and PP4), phorbol 12-myristate 13-acetate (PMA)/ionomycin stimulation (PC), and unstimulated negative controls (NC or baseline) in T1D and healthy controls. Abbreviations: PP, peptide pool; PC, positive control; NC, negative control; SSC, side scatter; T1D, type 1 diabetes; HC, healthy control.


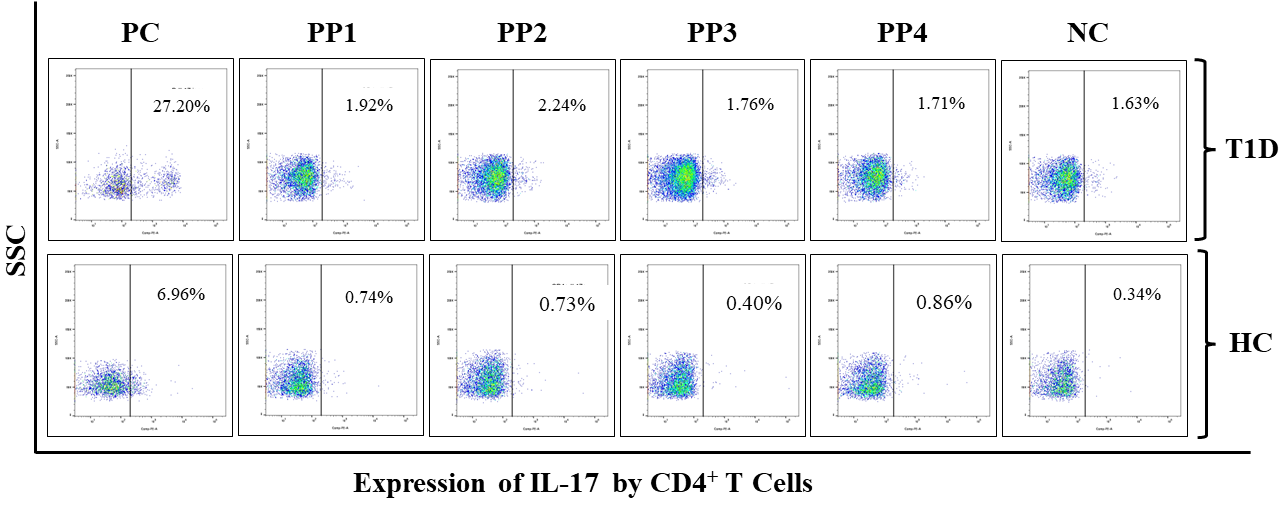
**Supplementary Figure 4**: Representative flow cytometric graphs showing CD4 T cells’ expression of IL-17 when cells were stimulated with four different peptide pools (PP1, PP2, PP3 and PP4), phorbol 12-myristate 13-acetate (PMA)/ionomycin stimulation (PC), and unstimulated negative controls (NC or baseline) in T1D and healthy controls. Abbreviations: PP, peptide pool; PC, positive control; NC, negative control; SSC, side scatter; T1D, type 1 diabetes; HC, healthy control.


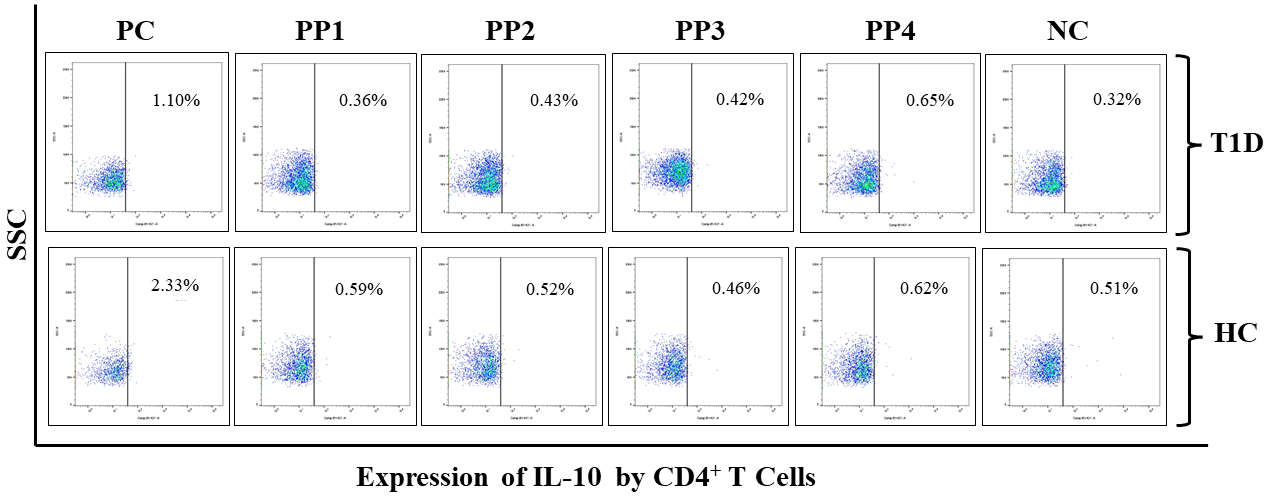
**Supplementary Figure 5**: Representative flow cytometric graphs showing CD4 T cells’ expression of IL-10 when cells were stimulated with four different peptide pools (PP1, PP2, PP3 and PP4), phorbol 12-myristate 13-acetate (PMA)/ionomycin stimulation (PC), and unstimulated negative controls (NC or baseline) in T1D and healthy controls. Abbreviations: PP, peptide pool; PC, positive control; NC, negative control; SSC, side scatter; T1D, type 1 diabetes; HC, healthy control.


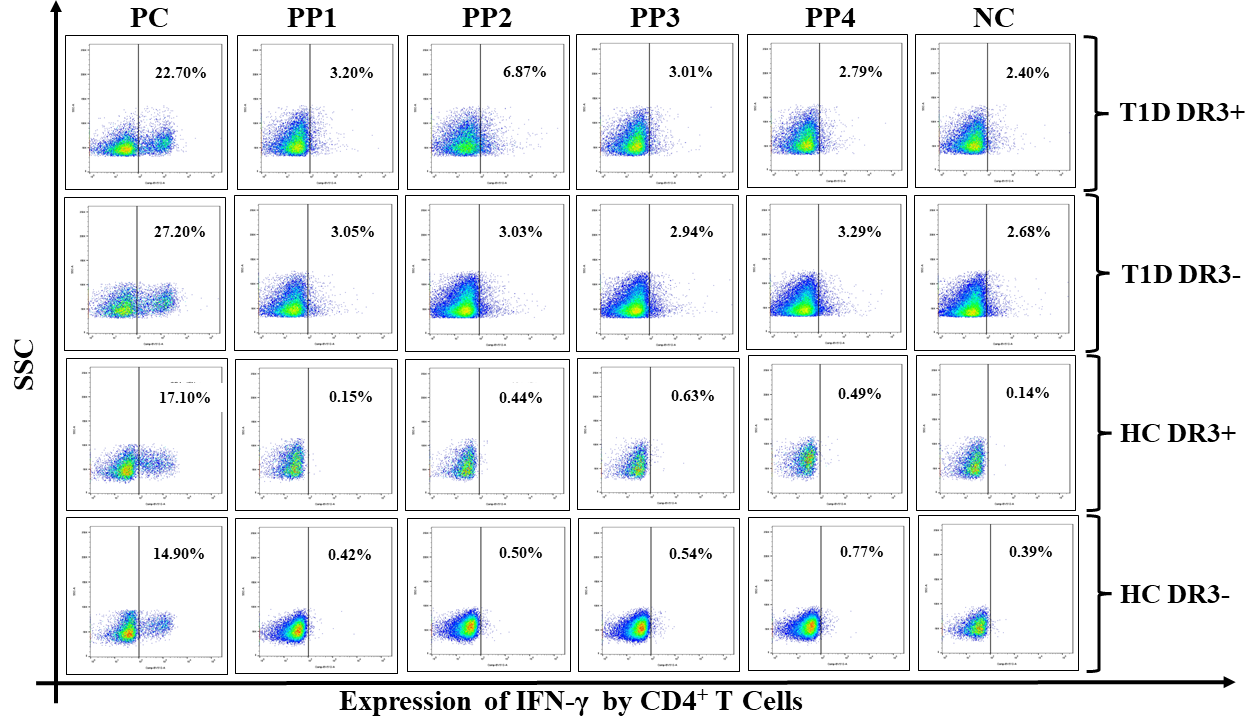
**Supplementary Figure 6**: Representative flow cytometric graphs showing CD4 T cells’ expression of IFN-γ when cells were stimulated with four different peptide pools (PP1, PP2, PP3 and PP4), phorbol 12-myristate 13-acetate (PMA)/ionomycin stimulation (PC), and unstimulated negative controls (NC or baseline) in HLA-DRB1*03-DQA1*05-DQB1*02 positive (DR3+) T1D patients, HLA-DRB1*03-DQA1*05-DQB1*02 negative (DR3–) T1D patients, HLA-DRB1*03-DQA1*05-DQB1*02 positive healthy controls and HLA-DRB1*03-DQA1*05-DQB1*02 negative healthy controls. Abbreviations: PP, peptide pool; PC, positive control; NC, negative control; SSC, side scatter; T1D, type 1 diabetes; HC, healthy controls.


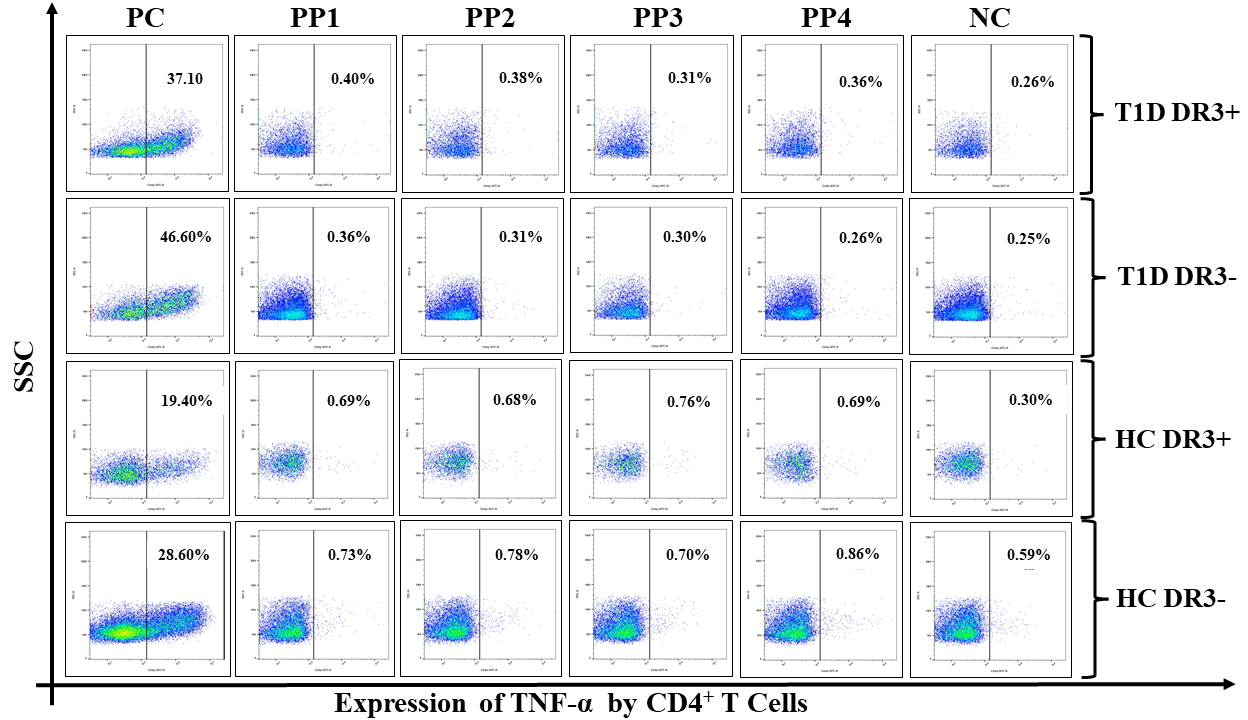
**Supplementary Figure 7**: Representative flow cytometric graphs showing CD4 T cells’ expression of TNF-α when cells were stimulated with four different peptide pools (PP1, PP2, PP3 and PP4), phorbol 12-myristate 13-acetate (PMA)/ionomycin stimulation (PC), and unstimulated cells (UC or baseline) in HLA-DRB1*03-DQA1*05-DQB1*02 positive (DR3+) T1D patients, HLA-DRB1*03-DQA1*05-DQB1*02 negative (DR3–) T1D patients, HLA-DRB1*03-DQA1*05-DQB1*02 positive healthy controls and HLA-DRB1*03-DQA1*05-DQB1*02 negative healthy controls. Abbreviations: PP, peptide pool; PC, positive control; NC, negative control; SSC, side scatter; T1D, type 1 diabetes; HC, healthy controls.


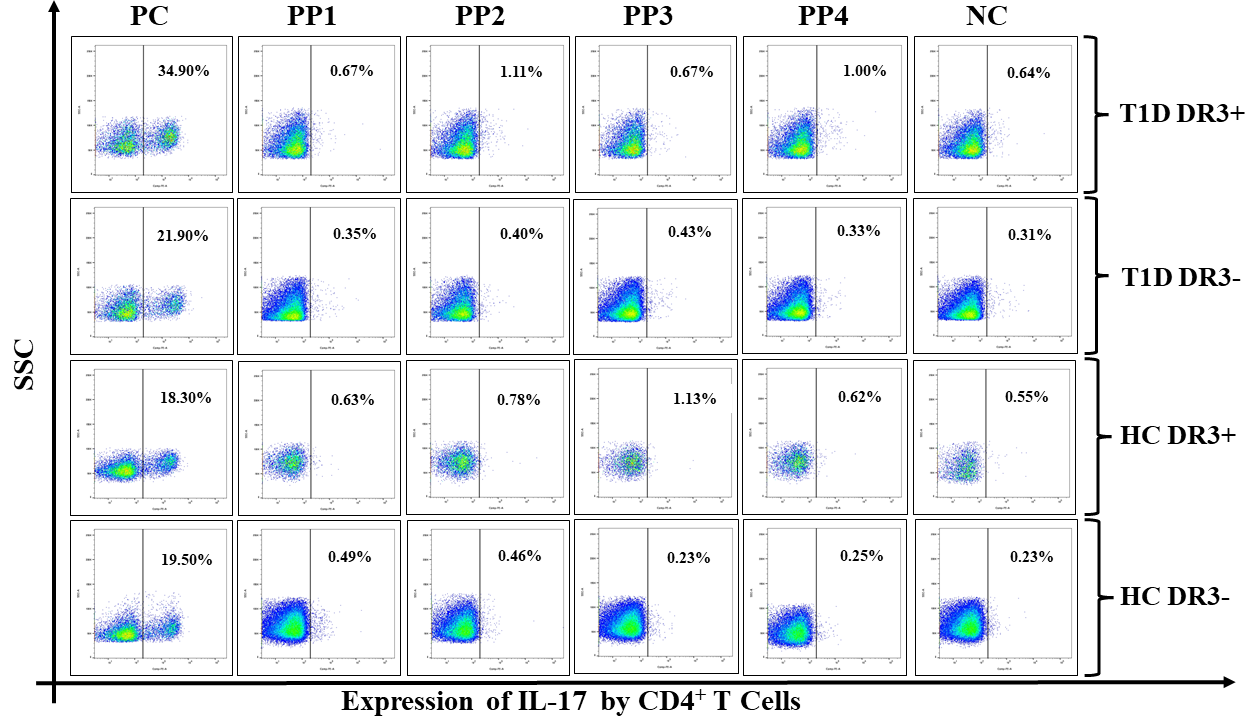
**Supplementary Figure 8**: Representative flow cytometric graphs showing CD4 T cells’ expression of IL-17 when cells were stimulated with four different peptide pools (PP1, PP2, PP3 and PP4), phorbol 12-myristate 13-acetate (PMA)/ionomycin stimulation (PC), and unstimulated negative controls (NC or baseline) in HLA-DRB1*03-DQA1*05-DQB1*02 positive (DR3+) T1D patients, HLA-DRB1*03-DQA1*05-DQB1*02 negative (DR3–) T1D patients, HLA-DRB1*03-DQA1*05-DQB1*02 positive healthy controls and HLA-DRB1*03-DQA1*05-DQB1*02 negative healthy controls. Abbreviations: PP, peptide pool; PC, positive control; NC, negative control; SSC, side scatter; T1D, type 1 diabetes; HC, healthy controls.


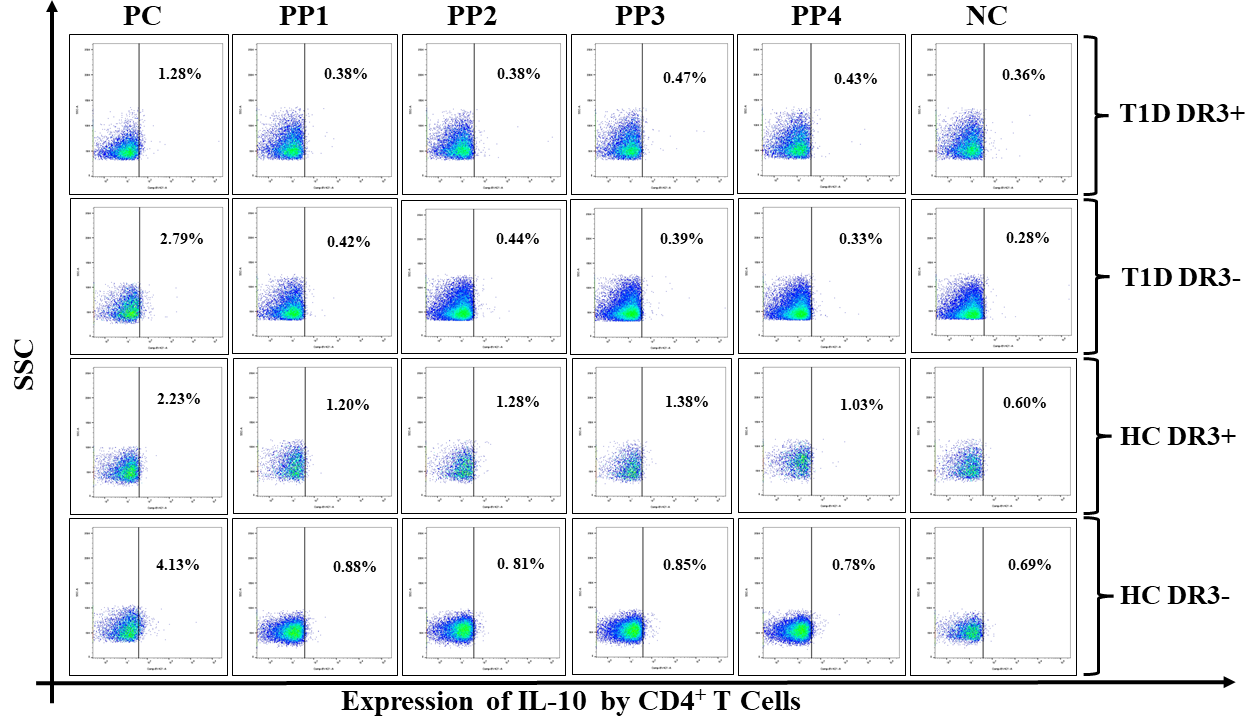
 **Supplementary Figure 9**: Representative flow cytometric graphs showing CD4 T cells’ expression of IL-10 when cells were stimulated with four different peptide pools (PP1, PP2, PP3 and PP4), phorbol 12-myristate 13-acetate (PMA)/ionomycin stimulation (PC), and unstimulated negative controls (NC or baseline) in HLA-DRB1*03-DQA1*05-DQB1*02 positive (DR3+) T1D patients, HLA-DRB1*03-DQA1*05-DQB1*02 negative (DR3–) T1D patients, HLA-DRB1*03-DQA1*05-DQB1*02 positive healthy controls and HLA-DRB1*03-DQA1*05-DQB1*02 negative healthy controls. Abbreviations: PP, peptide pool; PC, positive control; NC, negative control; SSC, side scatter; T1D, type 1 diabetes; HC, healthy controls.


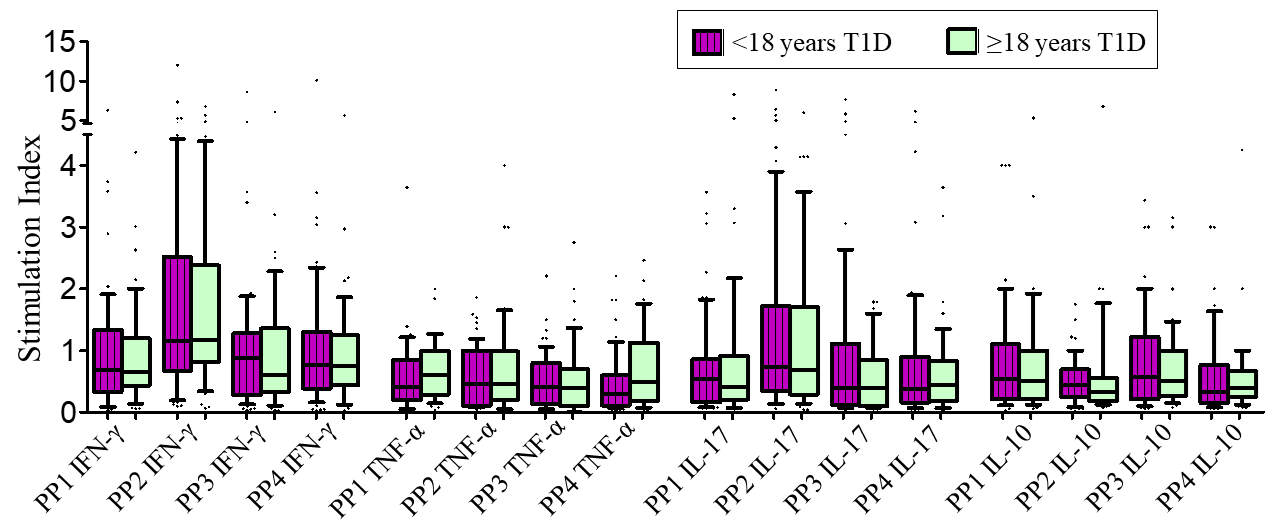
**Supplementary Figure 10**: Stimulation indices of CD4 T cells’ expression of IFN-γ, TNF-α, IL-17 and IL-10 when the cells were stimulated with four different GAD65 peptide pools (PP1, PP2, PP3 and PP4) in individuals clinically diagnosed with T1D before the age of 18 (<18) and those diagnosed with T1D on or after 18 years of age (≥18). The results are represented by box and whisker plots where boxes represent mean±SEM and whiskers represent 10-90 percentile data range. Abbreviations: PP, peptide pool; T1D, type 1 diabetes; SEM, standard error of mean.


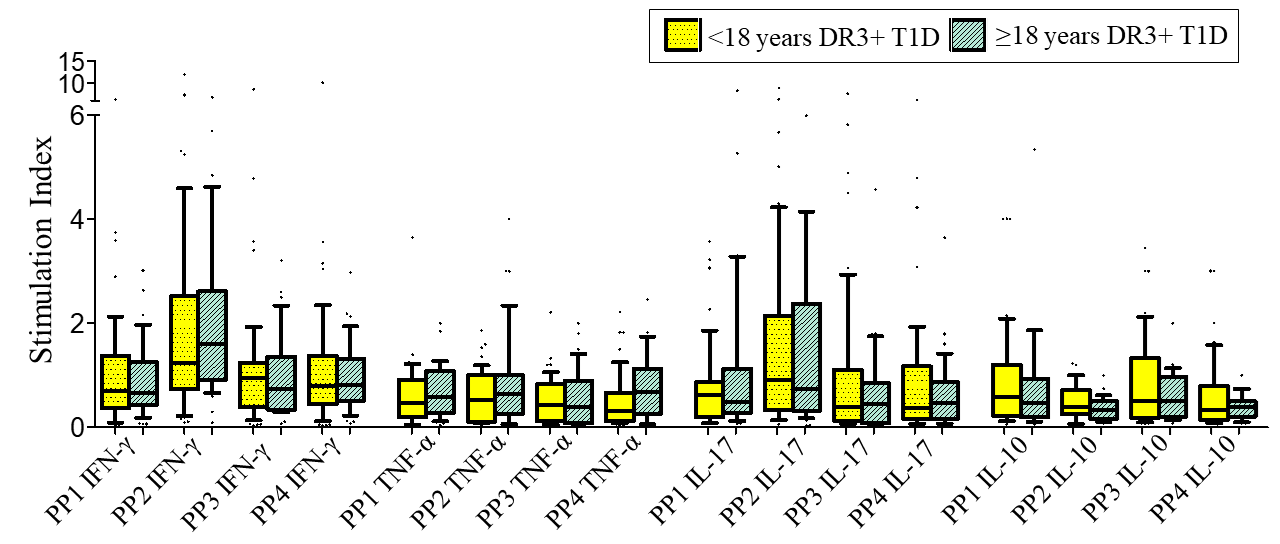
**Supplementary Figure 11**: Stimulation indices of CD4 T cells’ expression of IFN-γ, TNF-α, IL-17 and IL-10 when the CD4 T cells were stimulated with four different GAD65 peptide pools (PP1, PP2, PP3 and PP4) in HLA-DRB1*03-DQA1*05-DQB1*02 positive T1D patients with age at onset <18 years versus ≥18 years. The results are expressed as mean±SEM. Abbreviations: PP, peptide pool; T1D, type 1 diabetes; SEM, standard error of mean.


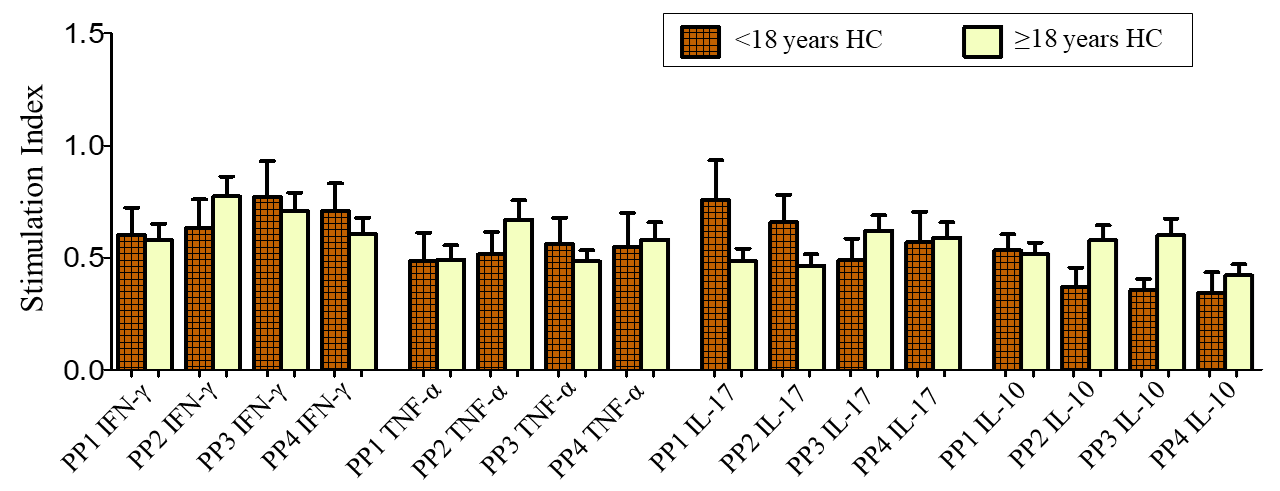
**Supplementary Figure 12**: Stimulation indices of IFN-γ, TNF-α, IL-17 and IL-10 cytokines expression by CD4 T cells when stimulated with four different GAD65 peptide pools (PP1, PP2, PP3 and PP4) in healthy controls with age at sampling <18 years and age at sampling ≥18 years. The results are expressed as mean±SEM. Abbreviations: PP, peptide pool; HC, healthy controls; SEM, standard error of mean.


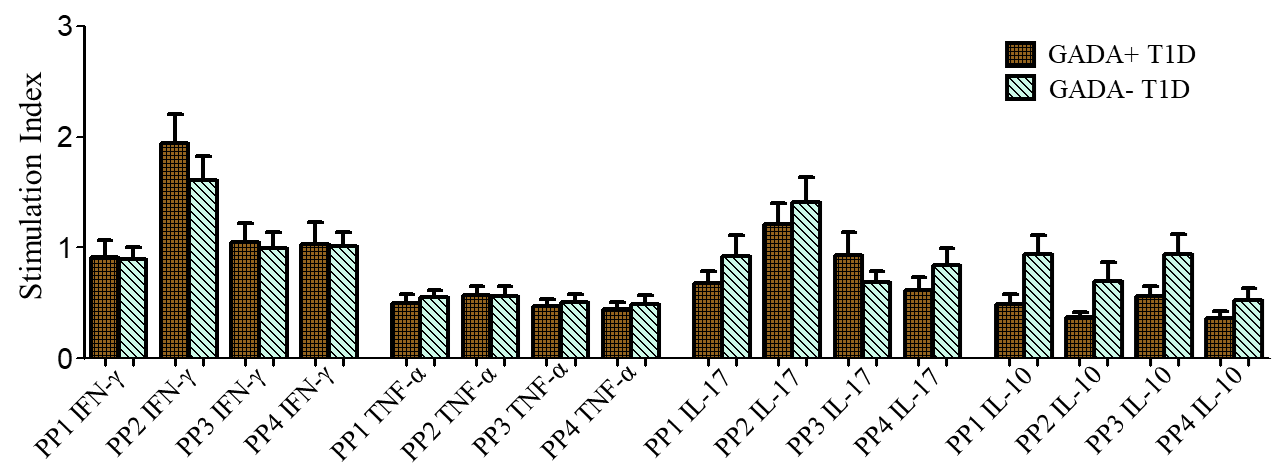
**Supplementary Figure 13:** Stimulation indices of IFN-γ, TNF-α, IL-17 and IL-10 cytokines expression by CD4 T cells when stimulated with four different GAD65 peptide pools (PP1, PP2, PP3 and PP4) in GADA positive and GADA negative T1D patients. The results are expressed as mean±SEM. Abbreviations: PP, peptide pool; GADA+, GAD65 autoantibody positive; GADA–, GAD65 autoantibody negative; T1D, type 1 diabetes; SEM, standard error of mean.


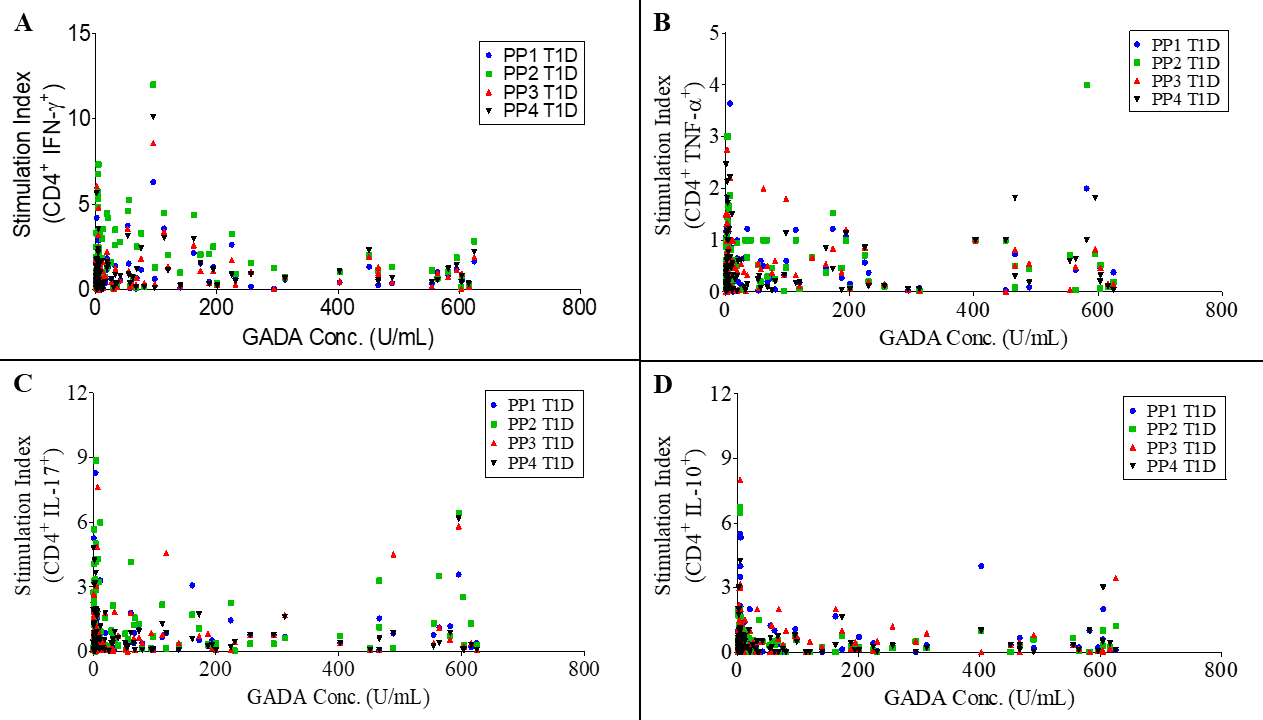
**Supplementary Figure 14:** GAD65 autoantibody titers versus stimulation indices (correlation) for cytokines IFN-γ (A), TNF-α (B), IL-17 (C) and IL-10 (D) expressed by T1D patients’ CD4 T cells when stimulated with four different GAD65 peptide pools (PP1, PP2, PP3 and PP4). The results are expressed as mean±SEM. Abbreviations: PP: peptide pool; GADA, GAD65 autoantibody; T1D, type 1 diabetes; SEM, standard error of mean.

*********************************

**Supplementary Material**

**Supplementary table 1**: Prevalence of GAD65 autoantibody in recently diagnosed (<2 years) versus long standing (≥2years) T1D patients and patients diagnosed with T1D before 18 years versus on or after 18 years of age

|  | **Duration of T1D** | | **Age at T1D Onset** | |
| --- | --- | --- | --- | --- |
|  | **<2 years**  **(N=23)** | **≥2years**  **(N=87)** | **<18 years**  **(N=63)** | **≥18 years**  **(N=47)** |
| **GADA Positive, N+ (% Freq.)** | 14 (60.87%) | 42 (48.28%) | 35 (55.56%) | 21 (44.68%) |
| **GADA & HLA-DRB1*03-DQA1*05-DQB1*02 Positive,**  **N+ (% Freq.)** | 12 (52.17%) | 36 (41.38%) | 31 (49.21%) | 18 (38.30%) |

Abbreviations: GADA, GAD65 autoantibody; Freq., frequency; N, total number of patients; T1D, type 1 diabetes.
